# “My wellbeing-their wellbeing “– An eHealth intervention for managing obesity in early care and education: Protocol for the Go NAPSACC Cares cluster randomized control trial

**DOI:** 10.1371/journal.pone.0286912

**Published:** 2023-07-07

**Authors:** Erik A. Willis, Regan Burney, Derek Hales, LeAndra O. Ilugbusi, Deborah F. Tate, Brooke Nezami, Emily C. Clarke, Renee H. Moore, Emma Mathews, Meredith Thompson, Brittany Beckelheimer, Dianne S. Ward

**Affiliations:** 1 Center for Health Promotion and Disease Prevention, University of North Carolina at Chapel Hill, Chapel Hill, North Carolina, United States of America; 2 Department of Nutrition, Gillings School of Global Public Health, University of North Carolina at Chapel Hill, Chapel Hill, North Carolina, United States of America; 3 Department of Epidemiology and Biostatistics, School of Public Health, Drexel University, Philadelphia, Pennsylvania, United States of America; Public Library of Science, UNITED KINGDOM

## Abstract

**Background:**

To fully leverage the potential of the early care and education (ECE) setting for childhood obesity prevention, initiatives must not intervene solely at the organizational level, but rather they should also address the health needs of the ECE workers. Workers suffer disproportionately high rates of obesity, and have reported low confidence in modeling and promoting healthy eating and activity behaviors. However, information regarding the effectiveness of improving ECE workers’ health behaviors or whether such improvements elicit meaningful change in the ECE environment and/or the children in their care is limited.

**Method:**

The proposed study will integrate a staff wellness intervention into a nationally recognized, ECE obesity prevention initiative (Go NAPSACC). Go NAPSACC+ Staff Wellness program will be assessed using a clustered randomized controlled trial including 84 ECE centers, 168 workers, and 672 2–5-year-old children. Centers will be randomly assigned to 1) standard “Go NAPSACC” or 2) Go NAPSACC+ Staff Wellness. Outcome measures will assess impact on dietary intake and PA behaviors of 2-5-year-old children at 6 months (primary aim) and 12 months. Secondarily, we will compare the impact of the intervention on centers’ implementation of healthy weight practices and the effect on ECE workers’ diet quality and PA at 6- and 12 months.

**Discussion:**

This trial expects to increase our understanding of how ECE worker’s personal health behaviors impact the health behaviors of the children in their care and the ECE environment.

**Trial registration:**

ClinicalTrials.gov: NCT05656807, registered on 19 December 2022. Protocol version 1.0, 22 March 2023.

## Introduction

Despite significant efforts to thwart the increasing incidence of obesity, rates have continued to climb over the past two decades [1–3] Once established, obesity is associated with a myriad of health issues including type 2 diabetes [4–6] cardiovascular disease [6–8] several cancers [9–11], lower quality of life [12] and high burden of medical costs (~$150 billion annually) [13,14]. Research shows that the ages between 0–5 years are a critical period in the development of obesity [15–17], and that childhood obesity is highly predictive of adult obesity [15,18]. Poor eating and physical activity habits, developed during childhood, are important causes of excessive weight gain [18]. Moreover, the environment to which young children are exposed, especially the adult-child relationships they experience, play a key role in shaping these habits that mediate risk of obesity [16,17,19]. As a result, early care and education (ECE) centers ─ and their staff ─ have been identified as critical partners in public health efforts to address today’s obesity epidemic.

Developed over 20 years ago, “Go NAPSACC” is one of the most widely implemented evidence-based childhood obesity prevention initiative [20–24]. Go NAPSACC’s user-friendly and interactive tools guide ECE directors through a five-step improvement process [20,25]. Go NAPSACC has been implemented in over 25 states and received national recognition as “best evidence for impact” on childhood obesity [26]. While Go NAPSACC is shown to be efficacious in improving organizational-level policies and practices around healthy eating and physical activity in ECE programs, the program is primarily delivered through ECE directors with little direct engagement of other workers (e.g., teachers, assistants, cooks). Research has shown that workers’ own eating and physical activity patterns reduce their confidence and ability to model and promote healthy lifestyle behaviors to children [27–30]. ECE workers specifically list their own lack of knowledge about nutrition and physical activity as critical barriers to obesity prevention efforts [31,32], including Go NAPSACC [33]. Untapped opportunities exist to address workers’ health that would both improve their own quality of life and create healthier environments for the children in their care.

Using Go NAPSACC, we have the opportunity to assess if a comprehensive early childhood obesity prevention initiative that also addresses the health of ECE workers elicits meaningful change in the ECE environment and health behaviors of children in their care. If successful, findings will provide a highly implementable, scalable, and sustainable strategy that would enhance the standard paradigm of early childhood obesity prevention initiatives. This manuscript presents the background, rationale, and design to be used in this study.

## Materials and methods

### Funding

The primary sponsor of this study is the National Institute of Diabetes and Digestive and Kidney Diseases as part of the United States National Institutes of Health, (R01DK128174).

### Design overview

The proposed study is a cluster-randomized trial with a six-month active intervention and a six-month no contact follow-up period. Centers in both arms will receive the standard Go NAPSACC program. The intervention arm will be enhanced with an ECE worker-level healthy lifestyle intervention that encourages healthy eating, increased physical activity, and weight management (Go NAPSACC+ Staff Wellness). The dual primary outcomes are child’s diet quality and physical activity between baseline and post-intervention (6 months); secondary outcomes include worker diet quality, physical activity, weight, and centers’ use of evidence-based healthy lifestyle practices. The assessment schedule is shown in Table 1. This study has been approved by the Institutional Review Board at the University of North Carolina-Chapel Hill and registered at Clinicaltrials.gov (NCT05656807, Protocol version 1.0, 22 March 2023).

**Table 1:** Assessment schedule.

	STUDY PERIOD									
	Pre-allocation	Allocation	Post-allocation							
TIMEPOINT	*-t*_*1*_	0	6-month active intervention						*t*_*1*_ *post intervention*	*t*_*2*_ *6-month follow-up*
**ENROLMENT:**										
*Eligibility screen*	X									
*Written informed consent*	X									
*Allocation*		X								
**INTERVENTIONS:**										
*Standard Go NAPSACC*			X	X	X	X	X	X		
*Go NAPSACC+ Staff Wellness*			X	X	X	X	X	X		
**ASSESSMENTS:**										
*Demographics*	X									
*Anthropometrics (Teacher only)*	X								X	X
*Child/Teacher Physical Activity*	X								X	X
*Child/Teacher Diet Quality*	X								X	X
*Center level Physical Activity/Nutrition Environment*	X								X	X

### Recruitment

A convenience sample of 84 ECE centers will be recruited in four cohorts across four years from central North Carolina. ECE centers will be identified from the NC Division of Child Development and Early Education website (ncchildcare.ncdhhs.gov/childcaresearch), a publicly available database of licensed ECE facilities. Recruitment efforts will incorporate a multi-phase approach to ensure the engagement of centers and center directors as well as workers and parents. Recruitment efforts within each county will start by identifying community organizations which have established working relationships with local ECE centers. These community partners will be asked to endorse study participation and help distribute study information through their existing communication channels (e.g., newsletters, emails, partner website, group meetings). These communications will be followed by personalized email and mailed invitations about the study directly to centers from the study team. Then the team will follow up with direct solicitation to center directors to gauge interest and confirm center eligibility. Eligible ECE centers must 1) be open year-round, 2) be licensed with no plans to close in the next two years, 3) have been in operation for at least one year, 4) have at least two classrooms serving children 2–5 years-old, 5) serve lunch, 6) have no history of Go NAPSACC participation in the past 6 months. Additionally, two ECE classroom teachers serving 2–5-year-old children and eight primary caregivers of children from each classroom must provide written consent to participate. Once initial interest and eligibility are confirmed, an in-person recruitment visit will be scheduled to introduce the study to ECE teachers, answer questions about participation, invite teacher participation, and conduct informed written consent procedures. Eligible ECE workers must 1) be 18 years-old or older, 2) be a teacher of a 2–5-year-old classroom, 3) be able to read English, and 4) not be pregnant, nor planning to become pregnant in the next year. Once a center and teachers agree to participate, we will work with them to recruit children from their center. A follow up visit will be scheduled to collect written consents and answer any questions from primary caregivers. Eligible children must 1) be in a classroom with a participating ECE teacher, 2) be 2–5 years old, and 3) consenting primary caregiver must be able to read English.

### Randomization

Centers will serve as the unit of randomization. Following baseline testing of each cohort, the centers will be randomized to either standard Go NAPSACC or Go NAPSACC+ Staff Wellness. Randomization tables will be created by the data manager using a permuted block approach, with block sizes of four. The project manager will use this randomization table to assign centers to each arm (1:1). Results of randomization will be restricted to only essential team members. Only the project manager and study staff helping to deliver the intervention will be aware of arm assignment. Investigators, study statistician, and data collectors will remain blinded.

### Intervention overview

#### Standard Go NAPSACC (0–6 months)

All participating ECE centers will implement the Go NAPSACC program. Go NAPSACC provides a well-known platform within the ECE setting that allows intervention materials and support to be available continuously; thus, participants can engage with intervention materials on their own schedule and progress at their own pace. Go NAPSACC offers a suite of interactive, online tools that guide center directors through a 5-step improvement process (described below) to increase use of healthy eating and physical activity evidence-based practices (https://gonapsacc.org/) [20–24].

### Standard Go NAPSACC theoretical model

The Go NAPSACC program is guided by Social Cognitive Theory (SCT) and the Social Ecological Model (SEM). A core concept in SCT is reciprocal determinism, which suggests that behavior is shaped by the dynamic and reciprocal interaction between personal, environmental, and behavioral factors. SCT also recognizes that behavioral capability, observational learning, reinforcement, expectancies, and self-efficacy influence behavior. SEM posits that health behaviors are shaped by individuals’ interactions with the physical and social environment, recognizing sources of influence coming from individual, interpersonal, organizational, community, and public policy levels. Go NAPSACC applies this to children in ECE by recognizing that their eating and physical activity behaviors will be shaped by this ECE environment, and the opportunities it offers to experience, learn about, and build skills needed to adopt healthy lifestyle habits.

### Standard Go NAPSACC components

Centers will have 6 months to complete two 3-month mini-cycles of Go NAPSACC’s five-step improvement process using the child nutrition and physical activity modules. The order of starting module will be randomized.

#### Director orientation

The director will lead the Go NAPSACC effort for their ECE center, helping ensure changes are applied center wide and not just to specific classrooms. The directors will take part in a 1-hour video conference orientation. The orientation will be provided by research staff using standardized materials, which will include a slide presentation with talking points and a user guide. The orientation will instruct center directors on how to use the Go NAPSACC online tools and guide them through the creation of a personalized account. Sessions will be recorded and 10% of sessions will be reviewed using a study specific fidelity checklist on a rolling basis to confirm the information was delivered as intended.

#### Access to Go NAPSACC tools

Directors will use their personalized account to access Go NAPSACC’s interactive, online tools that guide them through the 5-step improvement process: 1) **Assess** tools provide self-assessments to help directors reflect on their current practices. 2) **Plan** tools help directors use feedback from their self-assessment to select goals and create customized action plans to reach those goals. 3) **Take action** tools offer a library of tips and materials that help directors as they work through their action plan steps. 4) **Learn more** tools offer trainings to improve knowledge. 5) **Keep it up** tools encourage directors to retake the self-assessment to gauge progress, celebrate, and identify areas for future work.

#### Support for center implementation

Directors will receive monthly check-ins by video conference, telephone, or email from research staff. Check-ins will be used to encourage directors to complete at least one cycle of Go NAPSACC’s 5-step process for both the physical activity and nutrition modules, assist with goal setting and action planning, inquire about progress on goals, provide suggestions for meeting goals as needed, troubleshoot technical issues, and support navigation of the tips and materials library and trainings.

### Go NAPSACC+ staff wellness (0–6 months)

Centers randomized to the Go NAPSACC+ Staff Wellness arm will receive the traditional Go NAPSACC program (described above); in addition, consented ECE teachers will simultaneously receive an evidence-based healthy lifestyle intervention adapted from prior evidence-based weight management interventions [34–36]. Each teacher will have their own account (set up at orientation) through which to access materials that will support their adoption of evidence-based strategies for improving healthy eating and physical activity.

### Go NAPSACC+ Staff Wellness theoretical model

Healthy lifestyle intervention strategies, also guided by SCT, pull from national nutrition and physical activity recommendations [37–40] and behavior change techniques that increase intervention adherence and improve health behaviors. The intervention integrates techniques known to be most effective for changing diet and physical activity behaviors, including intention formation, goal setting (e.g., weight loss, weight maintenance), self-monitoring (diet, physical activity, weight), and personal feedback [41] These techniques will be enhanced by employing behavior shaping (gradually modifying physical activity goals), stimulus control (strategies to decrease cues for less desirable and increase cues for more desirable diet and physical activity behaviors), including cognitive strategies to increase self-efficacy and problem solving, such as identifying and developing plans for high-risk situations, making changes in the home and social environments, responding to stress with non-food techniques, and relapse prevention strategies to teach participants to recognize precursors and consequences of lapses.

### Go NAPSACC+ staff wellness components

#### ECE teacher orientation

ECE teachers will take part in a 1-hour in-person orientation session at their center, where research staff will introduce the healthy lifestyle intervention, including an overview of the Go NAPSACC+ Staff Wellness mobile web application that can be accessed on a desktop, phone, or tablet, a detailed description of the diet and physical activity goals, instructions on setting up and using the digital health devices, and reviewing program timeline and expectations. All participants will be provided a Fitbit Inspire 3^™^ activity tracker, an Aria Air smart scale (Fitbit, Inc., San Francisco, CA), and welcome items (e.g., microfiber towel, water bottle, exercise bands). Time will be allotted for demos, practice, questions, and troubleshooting (e.g., internet connectivity, web application data entry). Any technical issues will be resolved by contacting research staff by phone or email. A randomly selected 10% of sessions will be screened by a secondary staff member using a study specific fidelity checklist on a rolling basis to confirm the information was delivered as intended.

#### Go NAPSACC+ Staff Wellness website

**Fig 1:**
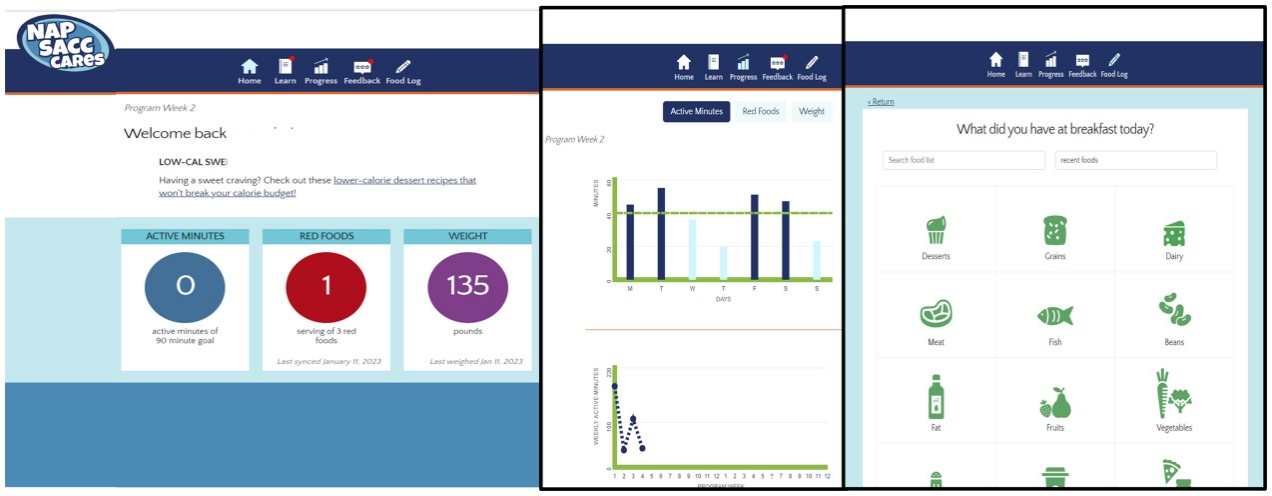
Sample Screenshots of the online web application.

The staff wellness mobile web application created for ECE workers will focus on personal healthy behavior change strategies and will include self-assessments, behavioral lessons, tailored feedback, food monitoring, and will link data from various mHealth devices (e.g., Fitbit, digital scales) to display on participants’ personal website accounts. Sample screenshots of the online applicaiton are shown in Fig 1.

#### Self-Assessment

Upon first login to the website participants will be prompted to complete a self-assessment, including questions about weight management goals (e.g., weight loss or weight maintenance), recent physical activity and eating behaviors, and recently used strategies and behaviors that an individual may have engaged in when trying to lose or maintain their weight. Responses from the self-assessment are used to create participants’ starting eating, physical activity, and their choice of a weight loss or weight maintenance goals will drive the messaging they receive throughout the program.

#### Dietary modification

Dietary recommendations will use an approach adapted from the Stoplight Diet (SLD). The SLD, originally developed by Epstein [42], is an effective weight loss strategy for both children [42–44] and adults [45–48]. Stoplight approaches categorize foods by energy content: green (low calories/high nutrients: consume freely), yellow (moderate calories/high nutrients: consume in moderation), and red (high calories/low nutrients: consume sparingly). Stoplight approaches may be easier for participants to understand and take less time than traditional calorie monitoring, particularly when asked to track only red foods. Tracking only red or high-calorie foods is a form of simplified or partial self-monitoring with demonstrated feasibility, acceptable adherence rates, and leads to clinically relevant weight losses [34,47,49]. In this study, lists and/or pictures of foods will be provided and will be color-coded for use in meal planning, shopping, and snacking. Teachers will receive a personalized daily red food goal based on their starting weight and weight change goal (e.g., a participant wanting to lose weight who is 250 lbs. will receive a daily red food goal of 3) and will be asked to reduce and record their servings of red foods in the food log section of the website.

#### Physical activity

We will prescribe a progressive moderate-intensity physical activity program (i.e., walking, jogging, biking, etc.) as recommended by the "physical activity Guidelines for Americans.” [38] Participants will receive a daily activity goal, with starting goals based on self-reported recent activity levels from the self-assessment: 0 minutes/ week = 10 minutes/day goal; 1–59 minutes/week. = 15 minutes /day goal, >60 minutes/week = 20 minutes/day goal. Attainment of weekly goals and progression will be based on data collected using Fitbit. If participants reach the equivalent of 5 times their daily activity goal in a given week, the following week, their daily goal will be increased by 5 minutes, up to a maximum of 60 minutes/day.

#### Self-monitoring

Participants will be asked to self-monitor red foods on the web application, physical activity using their Fitbit, and self-weigh daily using their smart scale. These data, which are synced to study servers in real-time, will be used for displaying participant progress, tailoring study messages, and for staff to assess fidelity of the program. *Diet*. Participants will log their red foods in the web application food log and view their dietary progress on the “Diet” page of the Go NAPSACC+ Staff Wellness web application. They can view the current day’s tally of red foods and beverages, in addition to a graph of totals over time. *Physical activity*. Participants will wear their Fitbit activity tracker and can also track activities manually in the Fitbit app when needed. They can view the current day’s activity minutes and graphs of progress over time on the “Activity” page of the web application. *Body weight*. Participants will weigh themselves using their smart scale which will sync their weight with the Fitbit app and study servers. They can view their most recent weight and overall weight progress on the “Weight” section of the web application.

#### Behavioral lessons

The Go NAPSACC+ Staff Wellness web application will unlock new lessons weekly for the first 12 weeks and then biweekly the next twelve (6 months total). Lessons will consist of written content focusing on reinforcing weight management strategies, including what strategies look like, why they are important, and practical tips for incorporating them into their everyday lives. Lessons will include topics such as portion sizes, reducing caloric beverages and red foods, emotional eating, behavioral strategies to increase physical activity, importance of sleep and stress management, stimulus control, sustaining changes, problem solving, and relapse prevention. Lessons will also include a Caregiver Corner that is specifically created for teachers working in the ECE setting. The Caregiver Corner will provide suggestions for incorporating lesson topics into their classroom. For example, a lesson about making healthy choices in social situations includes a Caregiver Corner section offering strategies for planning healthy classroom parties with children.

#### Tailored weekly feedback

Participants will receive weekly summaries of their progress on the web application. Participants self-monitoring data on diet, physical activity, and weight, along with their weight goal (loss or maintenance) will be used to tailor their feedback, which will include positive reinforcement for meeting goals and encouragement, tips, and specific behavior change techniques to promote future tracking and goal adherence.

#### Text messages

Participants will receive up to 5 text messages per week, which will include prompts to complete the lessons and review resource materials, messages that are tailored to their self-monitoring data to remind them to track or help them reach their eating, activity, or weight goals, and general tips for healthy behaviors in the childcare classroom.

### Secondary users

As per protocol two teachers from each center will be recruited and enrolled for all study components. However, at centers randomized to the Go NAPSACC+ Staff Wellness arm, all other workers will be invited to register as a secondary user. These users will not officially be enrolled in study outcome measures and will not receive Fitbit trackers or scales but will have access to the web application and its resources. They will be encouraged to manually log their activity and weight in the Fitbit app, will be able to view their progress on the study web application, and will receive text messages and tailored weekly feedback. The number of secondary users within each center will be recorded as part of process measures. For centers randomized to the control arm, once all study data has been collected at the 12 month follow up, any worker will be provided information about becoming a secondary user.

#### No contact follow-up (7–12 mos)

After the 6-month intervention period, all centers will continue to have access to the Go NAPSACC online tools and will be encouraged to continue additional cycles of the improvement process; however, the active engagement with research staff and their ongoing support will cease. Similarly, participants in the Go NAPSACC+ Staff Wellness arm will also continue to have access to the healthy lifestyle intervention suite of online tools and self-monitoring devices (i.e., Fitbit and smart scale); however, the active engagement of participants via reminders, tailored feedback, and motivational messages will cease.

### Measures

ECE workers (i.e., center directors, classroom teachers) and children will complete a series of assessments at three time points: baseline (month 0), post-intervention (month 6), and post no contact follow-up (month 12). Although the Go NAPSACC program will be implemented center-wide (affecting the environment of all children and ECE workers in each center), measurements will only be collected on children and ECE teachers formally enrolled in the study. These measures will be collected during a two-day on-site visit to the ECE center and supplemented with self-administered web-based surveys. This on-site visit will be conducted by data collectors who have undergone extensive training and certification on all measurement procedures and are blinded to study-arm assignment. Identical data collection protocols will be used at each timepoint. Detailed descriptions of measures and plans for analysis for each aim are provided below.

### Dietary quality

Children’s dietary intakes will be assessed using the Dietary Observation for ECE (DOCC). DOCC is a direct observation protocol developed by our team [50], used to assess foods consumed by children while at ECE. The DOCC collects detailed dietary data, including types of foods provided to children, amounts served, consumed, traded, spilled, and shared, and has been shown to be a reliable method (inter-rater r >0.98) for collecting observational dietary data in ECE centers [51]. Data collectors trained and certified in the DOCC protocol can assess up to three children at a time. The DOCC data will be entered into Nutrition Data System for Research (NDSR) to calculate children’s daily intake of food groups, calories, and nutrients. Using NDSR output, we will calculate Healthy Eating Index-2015 (HEI-2015) scores which assess conformance with dietary guidelines, providing a score ranging from 0 to 100, where scores closer to 100 indicate higher diet quality [52–54].

Teachers’ dietary intake will be assessed using food records collected and analyzed by the Automated Self-Administered 24-hour (ASA24) Dietary Assessment Tool developed by the National Cancer Institute. This tool has demonstrated acceptable psychometric properties [55–57] and has been validated in adults [58]. ASA24 is an automated web-based tool which allows multiple 24-hour recalls to be completed independently by participants using any internet enabled device. The website prompts participants to report detailed information about all the food and beverages consumed, using a multi-step process in which they record eating occasions, times, and add foods and beverages consumed. Teachers will record all food, beverages, and supplements consumed for 3 days (2 weekdays, 1 weekend day) within a 2-week period. Using ASA24 output, we will calculate HEI-2015 scores [52–54].

### Physical activity

Physical activity will be assessed via GT3X+ accelerometers (ActiGraph, Pensacola, FL) worn on participants’ non-dominant wrist for 24h/day for 7 days. For the proposed study, accelerometers will be programmed to sample acceleration at 40 Hz. On the morning of the first day of the center site visit, a data collector will fit each participant (teachers and children) with the accelerometer upon arrival at the center. ECE teachers and parents will be instructed on how to reattach and remove the accelerometer. Cut-points developed for preschool-aged children [59] and adults [60]. will be applied to accelerometer data, as appropriate, to calculate minutes spent in different levels of physical activity (total non-sedentary, sedentary, light, moderate, and vigorous).

### Anthropometrics

Anthropometrics will be collected in the morning of the onsite visit while participants are in light clothing with shoes removed. *Weight* will be measured to the nearest 0.1 kg using a Seca digital scale (calibrated quarterly; seca, Chino, CA). For children, weight will be an average of 2 measures within 0.3 pounds and for teachers within 1 pound. *Height* will be measured to the nearest 1/8 inch using a stadiometer (Shorr Productions, Olney, MD). For all participants height will be calculated as an average of 2 measures within 0.25-inch BMI (kg/m^2^) will be calculated from height and weight.

#### ECE center environment

Centers’ implementation of nutrition and physical activity best practices will be assessed with the Environment and Policy Assessment and Observation (EPAO) instrument [61]. The EPAO uses direct observation and review of written documents to capture environmental components of ECE: foods and beverages provided, feeding practices, feeding environment, menus, time provided for active play and outdoor play, indoor and outdoor play environment, teacher active play practices, screen availability, teacher screen practices, education and professional development, and policy. This measure has good inter-rater reliability [61] and sensitivity to change following interventions [62–64]. For EPAO data collection, each classroom will be observed for a full day. Data will be scored according to the EPAO scoring rubric, which first assesses compliance with nutrition and physical activity best practices, scoring each best practice using a 4-point scale (where higher scores indicate better compliance). Best practice compliance variables are then used to calculate environmental component scores that capture the various aspects of the nutrition and physical activity environment. Each environmental component score is calculated by averaging the relevant best practice compliance scores. Finally, the environmental component scores are summed to determine an overall nutrition and physical activity environment score.

### Process measures

As in our previous trial [65], the process evaluation will be guided by Steckler and Linnan [66] and the RE-AIM model framework [67]. As suggested by RE-AIM, we will measure reach (% who participate in each intervention component, engagement with website, self-monitoring of PA, diet and weight, and worker representativeness); *effectiveness* (assessed via aims); *adoption* (center level measure of uptake & representativeness); *implementation* (dose delivered; adherence to quality and fidelity of established protocols); and *maintenance* (teacher and center level durability of effects 6-months post-intervention) [67]. The most current guidelines for operationalizing these measures will be utilized [68]. Our previous process evaluation work also includes measuring *recruitment* (# approached, # eligible, # enrolled and costs per center & workers enrolled), and *responsiveness* (teacher satisfaction and use/engagement with intervention components). Process measures will allow us to understand study results more fully—why the intervention did/did not work, for whom and under what conditions the intervention was most/least effective, opportunities for program improvement, and the potential for dissemination/sustainability.

Structured interviews will be conducted by virtual conference call (e.g., zoom) in a 20% random sample of ECE directors and teachers from the Go NAPSACC+ Staff Wellness arm at 6 and 12 months to gather information that might be useful in improving the content or implementation of the intervention in ECE settings. Topics will include preference for intervention format, length, barriers to intervention components, suggestions to improve the intervention, overall satisfaction, including the diet and physical activity recommendations, online trainings, and engagement with research staff.

### Data management

Participants will be assigned an ID number as part of the screening process. All data collection forms and tables thereafter will use only this participant ID. Participant information (ID, eligibility/ineligibility, enrollment) will be stored in a database on UNC Center for Health Promotion and Disease Prevention’s secure server. This server is only accessible to approved project staff. The Project Manager will oversee quality checking of all data, creation of derived variables, development of a data dictionary and data user manual, and the creation of the final master de-identified dataset. This de-identified dataset will be saved on a shared drive on UNC’s secure server. Those wanting access to the analysis dataset will request permission from the principal investigator and project manager, who in turn will work with the data manager to extract the specific data requested.

### Statistical analysis

Our primary analyses will involve testing change in enrolled children’s minutes of non-sedentary activity and HEI scores between standard Go NAPSACC and Go NAPSACC+ Staff Wellness groups at 6 (primary outcome) and 12 months (secondary outcome). Using maximum likelihood methods, we will use multi-level linear mixed models (PROC MIXED) with repeated measures to estimate change at 6 and 12 months and to test for statistical differences across groups in changes over time. Models will include random effects for cluster to account for covariance between participants within the same center as well as fixed effects for time, trial arm, and time*arm interaction. A proper error covariance structure will be chosen based on model fit indicated by model likelihood, Akaike Information Criterion and Bayesian Information Criterion. To further explore the effect of the intervention, these analyses will be followed by sensitivity analyses that adjust for baseline variables distributed differently between intervention groups and examine completers only. Similar analyses will be completed for each secondary outcome including teacher physical activity, HEI scores, weight, as well as center physical activity and nutrition environments. While every effort will be made to avoid missing data, we expect some missing data due to attrition and nonresponse. Missing data patterns will be explored under various assumptions (e.g., missing completely at random, missing at random, or missing not at random) and presented in the outcomes paper.

### Power calculation

This trial is powered to detect between group difference (Standard Go NAPSACC vs. Go NAPSACC+ Staff Wellness) of 5 units in child HEI score and 1 minute per hour in non-sedentary physical activity. Based on intraclass correlation (ICC) from our preliminary work and those of others [69,70], we estimate ICC of 0.04 and 0.07 for HEI score and 0.07 and 0.10 for non-sedentary physical activity at the teacher- and center-levels, respectively and standard deviation of change in HEI score of 11 units [71,72] and non-sedentary physical activity of 3 minutes per hour. Furthermore, we calculated power for each of the two primary comparisons using a type I error rate of 2.5%, thus keeping overall Type I error rate at 0.05. Sample size was initially calculated using assumptions associated with non-sedentary physical activity knowing that the higher ICC would require a larger sample size. This sample size was then used to conduct a power analysis for HEI score. Given this, a sample size of 84 centers, with two 2-5-year-old classroom per center, and an average of 4 children per classroom, for a total of 672 children, would allow us to detect a clinically meaningful difference in non-sedentary physical activity with 88% power and HEI score with 99% power at α = 0.025 level of significance between the two study arms. Even with up to 20% attrition for centers and children, a completer’s only analysis will still provide 81% and 98% power for change in non-sedentary physical activity and HEI score, respectively, which is higher attrition than we expect based on our previous trials [62,69,73]

### Participant retention

Every effort will be made to avoid missing data; however, we expect some missing data due to attrition and nonresponse. If participants choose to withdraw from this study, all data collected up until the point of withdrawal will be retained, however no additional information will be collected unless they provide additional written permission for further data collection at the time of their withdrawal. To minimize attrition, a variety of incentives will be provided to encourage participation in the data collection process. Specifically, center directors will receive $100 per for participating in the 2-day onsite data collection visit at each timepoint. ECE teachers will receive a $75 incentive for completing outcome assessments at each timepoint and those participating in structured interviews will receive an additional $25. In addition to monetary incentives, small gifts valued at $50 per time point will be given to each participating classroom (e.g., magna-tiles, mindfulness map, wooden fruits/veggies) and child (e.g., books, bubbles, chalk). Teachers randomized into Go NAPSACC+ Staff Wellness will be allowed to keep the Fitbit, smart scale, and welcome items. Teachers in the control arm will receive $225 each as compensation for not receiving healthy lifestyle intervention and devices.

### Data safety monitoring

The intervention and measurement protocols pose minimal risk to participants. Given the low potential for risk of participant harm, this data safety monitoring (DSM) plan emphasizes close monitoring of the trial by the principal investigator, project manager, study statistician, and two DSM officers. Data reviews for accrual, adherence, and withdrawals will be reported to DSM officers quarterly and adverse events will be completed as they occur. DSM officers will review the stopping rules report regarding statistical power implications of drop outs/ missing data on a yearly basis. Suboptimal recruitment will be classified as falling into one of three zones–yellow, amber, and red. Red zone classification is the most severe of these categories and is defined as less than 25% of the benchmark at the 25% time point or the 50% time point, or less than 50% of the benchmark at the 75% time point.

### Trial status and study timeline

As of April 2023, the study team has completed development of the Staff Wellness website, finalized protocols and project materials, and has started recruiting. Intervention and data collection are expected to be completed by May 2026. Data analysis and paper writing will be completed by the end of January 2027. Any protocol modifications will be reported to the Institutional Review Board at the University of North Carolina-Chapel Hill and updated on Clinicaltrials.gov.

## Discussion

Initiation and evaluation of the Go NAPSACC Cares program is based on the premise that ECE workers are critical for the success of ECE-based early childhood obesity prevention initiatives, with the goal of improving ECE workers’ eating and activity behaviors such that these positive changes have a ripple effect onto child behaviors. The study is expected to include 84 ECE programs, 168 teachers, and 672 children followed over 12 months. All participating ECE programs will be exposed to the Go NAPSACC program and an implementation specialist for 6 months. In the staff wellness arm teachers will also receive a tailored diet and physical activity intervention during this 6-month period. During the maintenance period (months 7–12) all participants will maintain access to their respective program materials but engagement with research staff and interventionists will cease.

Information regarding the effectiveness of improving worker health behaviors or whether such improvements elicit meaningful change in the ECE environment and the children in their care is limited [73–78]. For example, no study included in a 2020 scoping review of interventions targeting the health status of the ECE workforce reported data on the impact on child outcomes and only 5 of 11 studies reported worker health outcomes. 79 Moreover, the majority of these studies are limited by small samples [74–77], no control groups [76,77], and lack of objective measures of physical activity, environment, and workplace supports [74,76,77]. One study published subsequent to the 2020 scoping review found workers’ self-reported increases in their own health status were associated with greater improvements in the classroom environment, however, no objective measures of workers’ health or child outcomes were collected [74]. Moreover, the low-intensity of these interventions is an additional methodologic concern as evidence suggests more intensive lifestyle interventions are needed to produce clinically meaningful change in health behaviors [39]. Due to the limited body of evidence available, and the potential health benefits to workers and the children in their care, the 2020 scoping review concluded there is a critical need to develop and evaluate ECE worker health promotion interventions [79]. Considering Go NAPSACC’s wide dissemination within ECE setting, it offers a natural platform to deliver a health promotion intervention for ECE workers. This addition will not only address the health needs of workers but could also improve their engagement in Go NASPACC’s program-wide efforts to implement evidence-based childhood obesity prevention practices.

To address gaps in the field, we considered several design features. These included: type of worker intervention, BMI inclusions, dietary strategies, and self-monitoring approaches. Comprehensive healthy lifestyles approach with a focus on weight management was chosen because it has been identified as a primary health concern of ECE workers [29,80] and our own research has shown a high desire for such interventions. We hypothesize that meeting these demands of the workers, via a Go NAPSACC+ Staff Wellness program, will improve the health status of the worker, boost instruction and modeling of healthy lifestyle behaviors in ECE settings, and may subsequently enhance Go NAPSACC’s effect on the ECE environment. We chose to include ECE workers with BMI ≥18.5 kg/m2. An estimated 88% of the ECE worker population are classified with overweight or obesity [28], therefore, the majority of workers will benefit from a weight management intervention. For individuals who are within the healthy BMI range (18.5–24.9 kg/m2) recommendations will be tailored to improving physical activity and diet quality for general health. Self-monitoring is associated with improved weight loss and maintenance [39,81,82]. Adherence to self-monitoring protocols is improved, and the probability of dropout is reduced in weight management trials using electronic compared with traditional pencil/paper records [83,84]. Thus, to improve the low compliance with self-monitoring seen in our previous trial [85] we chose the Fitbit (PA) and Bluetooth-tooth enabled scales (weight) as these commercially available technologies are easy to use, provide immediate feedback, and interface seamlessly with any Bluetooth-enabled device (e.g. smartphone, tablet computer). The stoplight approach to dietary changes, originally developed by Epstein [42], is an effective weight loss strategy for both children [42–44] and adults [34,45–47] as indicated by a Grade 1 ranking (strong, consistent, supporting evidence) from The Academy of Nutrition and Dietetics Evidence Analysis Library [48].

A unique feature of this study is the inclusion of the secondary users within each center. While per study protocol two 2–5-year-old classroom teachers from each center will be recruited and enrolled for all study components, we will allow access to the staff wellness program to all ECE workers (e.g., other teachers, assistant teachers, and additional staff) in centers randomized to the Go NAPSACC+ Staff Wellness arm. Gauging interest and use of the staff wellness online tool (e.g., logins, lessons completed, health behavior data entered) of staff not officially enrolled in the study will provide insight into pragmatic use of the program in the ECE setting. This information will add additional insight regarding the accessibility and sustainability for future implementation.

## Conclusion

In conclusion, Go NAPSACC Cares is a clustered randomized trial to evaluate if improving ECE care workers’ health behaviors elicit meaningful change in dietary and physical activity behaviors in 2-5-year-old preschool children in their care and of the ECE environment. The study will improve our understanding of how workers’ own health outcomes facilitate the impact of such initiatives. It will also provide a highly implementable, scalable, and sustainable strategy that would optimize obesity prevention in the ECE setting. The Go NAPSACC Cares study represents an important step forward to evaluate the potential impact ECE workers have on the care environment and children’s eating and physical activity behaviors.

## Supporting information

S1 File Original institutional review board approved protocol. (PDF)

## References

[1] Flegal KM, Carroll MD, Kit BK, Ogden CL. Prevalence of obesity and trends in the distribution of body mass index among US adults, 1999–2010. *JAMA*. 2012;307(5):491–497. doi: 10.1001/jama.2012.39 22253363

[2] Flegal KM, Carroll MD, Ogden CL, Johnson CL. Prevalence and trends in obesity among US adults, 1999–2000. *JAMA*. 2002;288(14):1723–1727. doi: 10.1001/jama.288.14.1723 12365955

[3] Hales CM, Fryar CD, Carroll MD, Freedman DS, Ogden CL. Trends in Obesity and Severe Obesity Prevalence in US Youth and Adults by Sex and Age, 2007–2008 to 2015–2016. *JAMA*. 2018;319(16):1723–1725. doi: 10.1001/jama.2018.3060 29570750 PMC5876828

[4] Mokdad AH, Bowman BA, Ford ES, Vinicor F, Marks JS, Koplan JP. The continuing epidemics of obesity and diabetes in the United States. *JAMA*. 2001;286(10):1195–1200. doi: 10.1001/jama.286.10.1195 11559264

[5] Mokdad AH, Ford ES, Bowman BA, et al. Prevalence of obesity, diabetes, and obesity-related health risk factors, 2001. *JAMA*. 2003;289(1):76–79. doi: 10.1001/jama.289.1.76 12503980

[6] Must A. Does overweight in childhood have an impact on adult health? *Nutrition reviews*. 2003;61(4):139. doi: 10.1301/nr.2003.apr.139-142 12795448

[7] Hubert HB, Feinleib M, McNamara PM, Castelli WP. Obesity as an independent risk factor for cardiovascular disease: a 26-year follow-up of participants in the Framingham Heart Study. *Circulation*. 1983;67(5):968–977. doi: 10.1161/01.cir.67.5.968 6219830

[8] Poirier P, Giles TD, Bray GA, et al. Obesity and cardiovascular disease: pathophysiology, evaluation, and effect of weight loss: an update of the 1997 American Heart Association Scientific Statement on Obesity and Heart Disease from the Obesity Committee of the Council on Nutrition, Physical Activity, and Metabolism. *Circulation*. 2006;113(6):898–918. doi: 10.1161/CIRCULATIONAHA.106.171016 16380542

[9] Chen Y, Wang X, Wang J, Yan Z, Luo J. Excess body weight and the risk of primary liver cancer: an updated meta-analysis of prospective studies. *Eur J Cancer*. 2012;48(14):2137–2145. doi: 10.1016/j.ejca.2012.02.063 22446023

[10] Genkinger JM, Spiegelman D, Anderson KE, et al. A pooled analysis of 14 cohort studies of anthropometric factors and pancreatic cancer risk. *Int J Cancer*. 2011;129(7):1708–1717. doi: 10.1002/ijc.25794 21105029 PMC3073156

[11] Sanfilippo KM, McTigue KM, Fidler CJ, et al. Hypertension and obesity and the risk of kidney cancer in 2 large cohorts of US men and women. *Hypertension*. 2014;63(5):934–941. doi: 10.1161/HYPERTENSIONAHA.113.02953 24637660 PMC4098147

[12] Ul-Haq Z, Mackay DF, Fenwick E, Pell JP. Meta-analysis of the association between body mass index and health-related quality of life among adults, assessed by the SF-36. *Obesity (Silver Spring)*. 2013;21(3):E322–327. 10.1002/oby.20107 23592685

[13] Kim DD, Basu A. Estimating the Medical Care Costs of Obesity in the United States: Systematic Review, Meta-Analysis, and Empirical Analysis. *Value Health*. 2016;19(5):602–613. doi: 10.1016/j.jval.2016.02.008 27565277

[14] Biener A, Cawley J, Meyerhoefer C. The Impact of Obesity on Medical Care Costs and Labor Market Outcomes in the US. *Clin Chem*. 2018;64(1):108–117. doi: 10.1373/clinchem.2017.272450 29097513

[15] Cunningham SA, Datar A, Narayan KMV, Kramer MR. Entrenched obesity in childhood: findings from a national cohort study. *Ann Epidemiol*. 2017;27(7):435–441. doi: 10.1016/j.annepidem.2017.05.016 28645567 PMC5550333

[16] Matthews E, Wei J, Cunningham S. Relationship between prenatal growth, postnatal growth and childhood obesity: a review. *European Journal of Clinical Nutrition*. 2017;71(8):919–930. doi: 10.1038/ejcn.2016.258 28247860

[17] Cunningham SA, Kramer MR, Narayan K. Incidence of childhood obesity in the United States. *N Engl J Med*. 2014;370:403–411. doi: 10.1056/NEJMoa1309753 24476431 PMC4017620

[18] Ward ZJ, Long MW, Resch SC, Giles CM, Cradock AL, Gortmaker SL. Simulation of growth trajectories of childhood obesity into adulthood. *N Engl J Med*. 2017;377:2145–2153. doi: 10.1056/NEJMoa1703860 29171811 PMC9036858

[19] Cunningham SA, Datar A, Narayan KV, Kramer MR. Entrenched obesity in childhood: findings from a national cohort study. *Annals of epidemiology*. 2017;27(7):435–441. doi: 10.1016/j.annepidem.2017.05.016 28645567 PMC5550333

[20] Ammerman AS, Ward DS, Benjamin SE, et al. An intervention to promote healthy weight: Nutrition and Physical Activity Self-Assessment for Child Care (NAP SACC) theory and design. *Prev Chronic Dis*. 2007;4(3):A67. 17572971 PMC1955393

[21] Battista RA, Oakley H, Weddell MS, Mudd LM, Greene J, West ST. Improving the physical activity and nutrition environment through self-assessment (NAP SACC) in rural area child care centers in North Carolina. *Preventive medicine*. 2014;67:S10–S16. doi: 10.1016/j.ypmed.2014.01.022 24495522

[22] Dinkel D, Dev D, Guo Y, et al. Improving the physical activity and outdoor play environment of family child care homes in Nebraska through go nutrition and physical activity self-assessment for child care. *Journal of Physical Activity and Health*. 2018;15(10):730–736. doi: 10.1123/jpah.2017-0411 29741448

[23] Ward DS, Vaughn AE, Mazzucca S, Burney R. Translating a child care based intervention for online delivery: development and randomized pilot study of Go NAPSACC. *BMC Public Health*. 2017;17(1):891. doi: 10.1186/s12889-017-4898-z 29162057 PMC5698966

[24] *QuickStats*: *Age-Adjusted Percentage of Adults Aged ≥20 Years Who Tried to Lose Weight During the Past 12 Months*, *by Sex—National Health and Nutrition Examination Survey*, *2007–2008 to 2015–2016*. 2018. 10.15585/mmwr.mm6741a10 PMC6193687 30335733

[25] NAPSACC. NAPSACC: Our History. https://gonapsacc.org/. Accessed August 5, 2016.

[26] Kenney E CA, Resch S, Giles C, Gortmaker S. The Cost-Effectiveness of Interventions for Reducing Obesity among Young Children through Healthy Eating, Physical Activity, and Screen Time. 2019.

[27] Snyder K, Hill M, Lee M, Crawford TN, Orlowski M. The Relationships Between Physical Health and Chronic Disease, Stress, and Resource Strain in Head Start Employees. *Workplace health & safety*. 2019:2165079919882952. 10.1177/2165079919882952 31735135

[28] Linnan L, Arandia G, Bateman LA, Vaughn A, Smith N, Ward D. The Health and Working Conditions of Women Employed in Child Care. *Int J Environ Res Public Health*. 2017;14(3):283. doi: 10.3390/ijerph14030283 28282940 PMC5369119

[29] Sharma S, Dortch KS, Byrd-Williams C, et al. Nutrition-related knowledge, attitudes, and dietary behaviors among head start teachers in Texas: a cross-sectional study. *Journal of the Academy of Nutrition and Dietetics*. 2013;113(4):558–562. doi: 10.1016/j.jand.2013.01.003 23415503 PMC3619413

[30] Whitaker RC, Becker BD, Herman AN, Gooze RA. Peer Reviewed: The Physical and Mental Health of Head Start Staff: The Pennsylvania Head Start Staff Wellness Survey, 2012. *Preventing chronic disease*. 2013;10. 10.5888/pcd10.130171 PMC3816599 24176085

[31] Hughes CC, Gooze RA, Finkelstein DM, Whitaker RC. Barriers to obesity prevention in Head Start. *Health Aff (Millwood)*. 2010;29(3):454–462. doi: 10.1377/hlthaff.2009.0499 20194987

[32] Sharma S, Dortch KS, Byrd-Williams C, et al. Nutrition-related knowledge, attitudes, and dietary behaviors among Head Start teachers in Texas: a cross-sectional study. *Journal of the Academy of Nutrition and Dietetics*. in press. doi: 10.1016/j.jand.2013.01.003 23415503 PMC3619413

[33] Martin SL, Martin MW, Cook B, Knaus R, O’Rourke K. Notes from the field: the evaluation of Maine Nutrition and Physical Activity Self-Assessment for Child Care (NAPSACC) experience. Eval Health Prof. 2015;38(1):140–145. doi: 10.1177/0163278714536032 24872443

[34] Nezami BT, Hurley L, Power J, Valle CG, Tate DF. A pilot randomized trial of simplified versus standard calorie dietary self-monitoring in a mobile weight loss intervention. *Obesity (Silver Spring)*. 2022;30(3):628–638. doi: 10.1002/oby.23377 35146942 PMC9469733

[35] Valle CG, Nezami BT, Tate DF. Designing in-app messages to nudge behavior change: Lessons learned from a weight management app for young adults. *Organizational Behavior and Human Decision Processes*. 2020;161:95–101.

[36] Wing RR, Tate DF, Espeland MA, et al. Innovative self-regulation strategies to reduce weight gain in young adults: the study of novel approaches to weight gain prevention (SNAP) randomized clinical trial. *JAMA internal medicine*. 2016;176(6):755–762. doi: 10.1001/jamainternmed.2016.1236 27136493 PMC5461816

[37] Health UDo, Services H. *Dietary guidelines for Americans 2015–2020*. Skyhorse Publishing Inc.; 2017.

[38] Piercy KL, Troiano RP, Ballard RM, et al. The physical activity guidelines for Americans. *Jama*. 2018;320(19):2020–2028. doi: 10.1001/jama.2018.14854 30418471 PMC9582631

[39] Jensen MD, Ryan DH, Apovian CM, et al. 2013 AHA/ACC/TOS guideline for the management of overweight and obesity in adults: a report of the American College of Cardiology/American Heart Association Task Force on Practice Guidelines and The Obesity Society. *Journal of the American College of Cardiology*. 2014;63(25 Pt B):2985–3023. doi: 10.1016/j.jacc.2013.11.004 24239920

[40] Donnelly JE, Blair SN, Jakicic JM, et al. American College of Sports Medicine Position Stand. Appropriate physical activity intervention strategies for weight loss and prevention of weight regain for adults. *Med Sci Sports Exerc*. 2009;41(2):459–471. doi: 10.1249/MSS.0b013e3181949333 19127177

[41] Michie S, Abraham C, Whittington C, McAteer J, Gupta S. Effective techniques in healthy eating and physical activity interventions: a meta-regression. *Health Psychol*. 2009;28(6):690–701. doi: 10.1037/a0016136 19916637

[42] Epstein LH, Squires S. *Stoplight diet for children*. Little, Brown; 1988.

[43] Epstein LH, Valoski A, Wing RR, McCurley J. Ten-year follow-up of behavioral, family-based treatment for obese children. *JAMA*. 1990;264(19):2519–2523. 2232019

[44] Epstein LH, Valoski A, Wing RR, McCurley J. Ten-year outcomes of behavioral family-based treatment for childhood obesity. *Health Psychol*. 1994;13(5):373–383. doi: 10.1037//0278-6133.13.5.373 7805631

[45] Ptomey LT, Saunders RR, Saunders M, et al. Weight management in adults with intellectual and developmental disabilities: A randomized controlled trial of two dietary approaches. *J Appl Res Intellect Disabil*. 2018;31 Suppl 1:82–96. doi: 10.1111/jar.12348 28332246

[46] Kim KH, Chung BY, Byun HS. The effects of weight control program on body composition, blood pressure, serum lipid and self-regulation behavior in obese college women. *J Korean Acad Adult Nurs*. 2007;19(3):339.

[47] Nezami BT, Ward DS, Lytle LA, Ennett ST, Tate DF. A mHealth randomized controlled trial to reduce sugar-sweetened beverage intake in preschool-aged children. *Pediatr Obes*. 2018;13(11):668–676. doi: 10.1111/ijpo.12258 29119719

[48] Myers EF. ADA Evidence Analysis Library. *Journal of the American Dietetic Association*. 2005;105(5 Suppl 1):S79. doi: 10.1016/j.jada.2005.03.029 15867901

[49] Tate DF, Quesnel DA, Lutes L, et al. Examination of a partial dietary self-monitoring approach for behavioral weight management. *Obes Sci Pract*. 2020;6(4):353–364. doi: 10.1002/osp4.416 32874670 PMC7448156

[50] Ward D, Hales D, Haverly K, et al. An instrument to assess the obesogenic environment of child care centers. *Am J Health Behav*. 2008;32(4):380–386. doi: 10.5555/ajhb.2008.32.4.380 18092898

[51] Ball SC, Benjamin SE, Ward DS. Development and reliability of an observation method to assess food intake of young children in child care. *Journal of the American Dietetic Association*. 2007;107(4):656–661. doi: 10.1016/j.jada.2007.01.003 17383271

[52] Guenther PM, Casavale KO, Reedy J, et al. Update of the Healthy Eating Index: HEI-2010. *J Acad Nutr Diet*. 2013;113(4):569–580. doi: 10.1016/j.jand.2012.12.016 23415502 PMC3810369

[53] Guenther PM, Kirkpatrick SI, Reedy J, et al. The Healthy Eating Index-2010 is a valid and reliable measure of diet quality according to the 2010 Dietary Guidelines for Americans. *J Nutr*. 2014;144(3):399–407. doi: 10.3945/jn.113.183079 24453128 PMC3927552

[54] Krebs-Smith SM, Pannucci TE, Subar AF, et al. Update of the Healthy Eating Index: HEI-2015. *J Acad Nutr Diet*. 2018;118(9):1591–1602. doi: 10.1016/j.jand.2018.05.021 30146071 PMC6719291

[55] National Cancer Institute. Automated Self-Administered 24-Hour (ASA24^®^) Dietary Assessment Tool. U.S. Department of Health and Human Services. https://epi.grants.cancer.gov/asa24/. Published 2017. Accessed.

[56] Kirkpatrick SI, Subar AF, Douglass D, et al. Performance of the Automated Self-Administered 24-hour Recall relative to a measure of true intakes and to an interviewer-administered 24-h recall. *Am J Clin Nutr*. 2014;100(1):233–240. doi: 10.3945/ajcn.114.083238 24787491 PMC4144101

[57] Park Y, Dodd KW, Kipnis V, et al. Comparison of self-reported dietary intakes from the Automated Self-Administered 24-h recall, 4-d food records, and food-frequency questionnaires against recovery biomarkers. *Am J Clin Nutr*. 2018;107(1):80–93. doi: 10.1093/ajcn/nqx002 29381789 PMC5972568

[58] Mitchell DC, Cheng FW, Still CD, Jensen GL. A Validation of Automated Self-Administered 24-Hour Dietary Recalls (ASA24) Relative to Interviewer-Administered Recalls using the Nutrition Data System for Research (NDSR). *The FASEB Journal*. 2016;30(1_supplement):43.43–43.43.

[59] Evenson KR, Catellier DJ, Gill K, Ondrak KS, McMurray RG. Calibration of two objective measures of physical activity for children. *J Sports Sci*. 2008;26(14):1557–1565. doi: 10.1080/02640410802334196 18949660

[60] Troiano RP, Berrigan D, Dodd KW, Masse LC, Tilert T, McDowell M. Physical activity in the United States measured by accelerometer. *Med Sci Sports Exerc*. 2008;40(1):181–188. doi: 10.1249/mss.0b013e31815a51b3 18091006

[61] Ward D, Hales D, Haverly K, et al. An instrument to assess the obesogenic environment of child care centers. *American Journal of Health Behavior*. 2008;32(4):380–386. doi: 10.5555/ajhb.2008.32.4.380 18092898

[62] Ward DS, Benjamin SE, Ammerman AS, Ball SC, Neelon BH, Bangdiwala SI. Nutrition and physical activity in child care: results from an environmental intervention. *Am J Prev Med*. 2008;35(4):352–356. doi: 10.1016/j.amepre.2008.06.030 18701236

[63] Lyn R, Maalouf J, Evers S, Davis J, Griffin M. Nutrition and physical activity in child care centers: the impact of a wellness policy initiative on environment and policy assessment and observation outcomes, 2011. *Prev Chronic Dis*. 2013;10:E83. doi: 10.5888/pcd10.120232 23701720 PMC3670649

[64] Benjamin Neelon SE, Taveras EM, Ostbye T, Gillman MW. Preventing obesity in infants and toddlers in child care: results from a pilot randomized controlled trial. *Matern Child Health J*. 2014;18(5):1246–1257. doi: 10.1007/s10995-013-1359-x 24065371 PMC3965661

[65] Linnan L, Vaughn A, Smith F, et al. Results of Caring and Reaching for Health (CARE): A Cluster-Randomized Controlled Trial Assessing a Worksite Wellness Intervention for Child Care Staff. Under Review. 10.1186/s12966-020-00968-x PMC7227251 32414381

[66] Steckler A, Linnan L. *Process Evaluation for Public Health Intervention and Research*. San Francisco: Jossey-Bass Publishers, Inc.; 2002.

[67] Glasgow RE, Vogt TM, Boles SM. Evaluating the public health impact of health promotion interventions: the RE-AIM framework. *Am J Public Health*. 1999;89(9):1322–1327. doi: 10.2105/ajph.89.9.1322 10474547 PMC1508772

[68] Institute NC. Reach Effectiveness Adoption Implementation Maintenance (RE-AIM). U.S. National Institutes of Health. http://cancercontrol.cancer.gov/is/reaim/. Published 2012. Updated January 10. Accessed May 29, 2012.

[69] Ward DS, Vaughn AE, Bangdiwala KI, et al. Integrating a family-focused approach into child obesity prevention: rationale and design for the My Parenting SOS study randomized control trial. *BMC Public Health*. 2011;11:431. doi: 10.1186/1471-2458-11-431 21639940 PMC3123597

[70] Hales D CP, McWilliams C, Vaughn A, Ward DS. Do childcare center policies relate to the physical activity levels of children?. *Oral presentation at the annual meeting of the International Society for Behavioral Nutrition and Physical Activity (ISBNPA)*, *Minneapolis*, *MN*. 2010.

[71] Ball SC, Benjamin SE, Ward DS. Dietary intakes in North Carolina child-care centers: are children meeting current recommendations? *Journal of the American Dietetic Association*. 2008;108(4):718–721. doi: 10.1016/j.jada.2008.01.014 18375233

[72] Vitolo MR, Rauber F, Campagnolo PD, Feldens CA, Hoffman DJ. Maternal dietary counseling in the first year of life is associated with a higher healthy eating index in childhood. *J Nutr*. 2010;140(11):2002–2007. doi: 10.3945/jn.110.125211 20844187

[73] Linnan LA, Vaughn AE, Smith FT, et al. Results of caring and reaching for health (CARE): a cluster-randomized controlled trial assessing a worksite wellness intervention for child care staff. *Int J Behav Nutr Phys Act*. 2020;17(1):64. doi: 10.1186/s12966-020-00968-x 32414381 PMC7227251

[74] Chuang RJ, Cox JN, Mincemoyer CC, Sharma SV. A Pilot Randomized Controlled Trial of a Nutrition and Dietary Intervention for Early Care and Education Providers. *J Sch Health*. 2020;90(11):859–868. doi: 10.1111/josh.12951 32959370

[75] Esquivel MK, Nigg CR, Fialkowski MK, Braun KL, Li F, Novotny R. Influence of Teachers’ Personal Health Behaviors on Operationalizing Obesity Prevention Policy in Head Start Preschools: A Project of the Children’s Healthy Living Program (CHL). *J Nutr Educ Behav*. 2016;48(5):318–325 e311. 27169640 10.1016/j.jneb.2016.02.007 PMC5496712

[76] Gosliner WA, James P, Yancey AK, Ritchie L, Studer N, Crawford PB. Impact of a worksite wellness program on the nutrition and physical activity environment of child care centers. *Am J Health Promot*. 2010;24(3):186–189. doi: 10.4278/ajhp.08022719 20073385

[77] Herman A, Nelson BB, Teutsch C, Chung PJ. "Eat Healthy, Stay Active!": a coordinated intervention to improve nutrition and physical activity among Head Start parents, staff, and children. *Am J Health Promot*. 2012;27(1):e27–36. doi: 10.4278/ajhp.110412-QUAN-157 22950932

[78] Ward DS, Vaughn AE, Burney RV, et al. Keys to healthy family child care homes: Results from a cluster randomized trial. *Preventive Medicine*. 2020;132:105974. doi: 10.1016/j.ypmed.2019.105974 31899253 PMC8091030

[79] Lessard LM, Wilkins K, Rose-Malm J, Mazzocchi MC. The health status of the early care and education workforce in the USA: a scoping review of the evidence and current practice. *Public Health Rev*. 2020;41(1):2. doi: 10.1186/s40985-019-0117-z 31934495 PMC6950818

[80] Vaughn AE, Martin CL, Ward DS. What matters most-what parents model or what parents eat? *Appetite*. 2018;126:102–107. doi: 10.1016/j.appet.2018.03.025 29604319 PMC5971159

[81] Zheng Y, Burke LE, Danford CA, Ewing LJ, Terry MA, Sereika SM. Patterns of self-weighing behavior and weight change in a weight loss trial. *International journal of obesity (2005)*. 2016;40(9):1392–1396. doi: 10.1038/ijo.2016.68 27113642

[82] Zheng Y, Terry MA, Danford CA, et al. Experiences of Daily Weighing Among Successful Weight Loss Individuals During a 12-Month Weight Loss Study. *Western journal of nursing research*. 2018;40(4):462–480. doi: 10.1177/0193945916683399 28322640

[83] Acharya SD, Elci OU, Sereika SM, Styn MA, Burke LE. Using a personal digital assistant for self-monitoring influences diet quality in comparison to a standard paper record among overweight/obese adults. *Journal of the American Dietetic Association*. 2011;111(4):583–588. doi: 10.1016/j.jada.2011.01.009 21443993 PMC3406749

[84] Semper HM, Povey R, Clark-Carter D. A systematic review of the effectiveness of smartphone applications that encourage dietary self-regulatory strategies for weight loss in overweight and obese adults. *Obesity reviews*: *an official journal of the International Association for the Study of Obesity*. 2016;17(9):895–906. doi: 10.1111/obr.12428 27192162

[85] Fossdal TS, Kippe K, Handegård BH, Lagestad P. “Oh oobe doo, I wanna be like you” associations between physical activity of preschool staff and preschool children. *PloS one*. 2018;13(11). 10.1371/journal.pone.0208001 PMC6264855 30496229

