# Supplementary material for: “My wellbeing-their wellbeing “– An eHealth intervention for managing obesity in early care and education: Protocol for the Go NAPSACC Cares cluster randomized control trial
Source: PLoS One. 2023 Jul 7;18(7):e0286912. doi: 10.1371/journal.pone.0286912 (PMC10328321; doi:10.1371/journal.pone.0286912)
Supplement: S1 File — (PDF) [file pone.0286912.s001.pdf]

Post Approval Submissions

Modification Information

To modify an approved study, edit the individual answers that make up the application. The questions below are intended solely for the IRB to have a summary statement of your requested action. The modifications cannot be processed until the actual changes have been made throughout the application.

1. Provide a brief non-technical summary of any changes you will be making to the study (i.e., study application, project personnel, and/or study documents.) The text you enter here will be reproduced in the IRB approval document, and should contain the details that you and/or your sponsor find relevant (e.g., master protocol/amendment version number and date). Typical summaries are 50-100 words. PLEASE NOTE: THIS SECTION MAY BE EDITED BY THE IRB FOR CLARITY OR LENGTH.

   We are adding a new research assistant, Brittany Beckelheimer to UNC personnel. Additionally, per request of the IRB we have uploaded forward facing intervention materials for both intervention arms. Go NAPSACC arm documentation includes orientation slides, self-assessments for physical activity and child nutrition from the Go NAPSACC website, and a sample training. Go NAPSACC Enhanced arm documentation includes orientation slides and screenshots from the NAPSACC Cares website.

2. Is this study in Data Analysis only (i.e. enrollment, intervention and follow-up are complete)?

   No

Total number of subjects enrolled to date:

43

Is this study currently open to the enrollment of new subjects?

Yes

Total number of subjects actively participating (i.e., Total number of subjects involved in the interventional part of this study. If the study is limited to data collection (e.g., surveys, questionnaires, collection of data from existing records), enter ‘0’:

0

3. Do you have plans to re-consent subjects as a result of this modification?

   No

4. Is this modification being submitted in response to Promptly Reportable Information?

   No

5. Have the risks as described in A.6., consent form, or any other study document changed? This may include new risks not previously listed, changes in frequency of known risks, or removal of previously listed risks.

   No

Continuing with Modifications

Click the "save and continue" button to access your existing application.
You may make any changes to the application that you are requesting at this time.

General Information

1. General Information

1. Project Title

   My weight-their weight: eHealth intervention for managing obesity in child care settings

2. **Brief Summary**. Provide a **brief non-technical description** of the study, which will be used in IRB documentation as a description of the study. Typical summaries are 50-100 words. Please reply to each item below, retaining the subheading labels already in place, so that reviewers can readily identify the content. PLEASE NOTE: THIS SECTION MAY BE EDITED BY THE IRB FOR CLARITY OR LENGTH.

   Purpose: The proposed 12-month study will use a cluster randomized controlled trial to evaluate if improving child care providers' health behaviors elicit meaningful change in dietary and PA behaviors in 2-5-year-old preschool children in their care and the child care environment.

   Participants: The study sample will include 84 child care centers (168 providers [=18 years old] and 672 children [2-5 years old]) which will be randomized (by center, 1:1).

   Procedures (methods): Centers will be randomized to receive either standard Go NAPSACC or Go NAPASACC plus a provider weight management component (Go NAPSACC Enhanced). Centers in both arms will receive the standard Go NAPSACC's program. The Go NAPSACC Enhanced arm will add a child care provider-level weight management intervention that encourages a reduced energy diet (stop light diet [SLD]), increased PA, and regular weight monitoring. We will use commercially available applications for self-monitoring PA (Fitbit Inspire 3) and weight (Aria wireless scales). The primary outcomes measures will assess children's diet quality and PA between baseline and post-intervention; secondary outcomes include provider weight, diet quality, and PA, and centers' use of evidence-based healthy weight practices. All measures will be collected at baseline, post-intervention (6 months), and maintenance (12 months post, no contact).

2. Project Personnel

1. Will this project be led by a STUDENT (undergraduate, graduate) or TRAINEE (resident, fellow, postdoc), working in fulfillment of requirements for a University course, program or fellowship?

   No

2. List all project personnel beginning with principal investigator, followed by faculty advisor, co-investigators, study coordinators, and anyone else who has contact with subjects or identifiable data from subjects.

   - List ONLY those personnel for whom this IRB will be responsible; do NOT include collaborators who will remain under the oversight of another IRB **for this study**.
   - If this is Community Based Participatory Research (CBPR) or you are otherwise working with community partners (who are not functioning as researchers), you may not be required to list them here as project personnel; consult with your IRB.
   - If your extended research team includes multiple individuals with limited roles, you may not be required to list them here as project personnel; consult with your IRB.

   The table below will access campus directory information; if you do not find your name, your directory listing may need to be updated.

Liaison	Last Name	First Name	Department Name	Role	
University of North Carolina at Chapel Hill (UNC-CH)					
	Willis	Erik	Center for Health Promotion and Disease Prevention	Principal Investigator	view
	Nezami	Brooke	Nutrition	Co-Investigator	view
	Tate	Deborah	Health Behavior	Co-Investigator	view
	Ward	Dianne	Nutrition	Co-Investigator	view
★	Burney	Regan	Center for Health Promotion and Disease Prevention	Study Coordinator	view
	Beckelheimer	Brittany	Center for Health Promotion and Disease Prevention	Research Assistant	view
	Clarke	Emily	Center for Health Promotion and Disease Prevention	Research Assistant	view
	Ilugbusi	LeAndra	Center for Health Promotion and Disease Prevention	Research Assistant	view

	Mathews	Emma	Center for Health Promotion and Disease Prevention	Research Assistant	view
	Rodriguez	Katelyn	Nutrition	Research Assistant	view
	Thompson	Meredith	Center for Health Promotion and Disease Prevention	Research Assistant	view
	Vasa	Riya	Thurston Arthritis Research Center	Research Assistant	view
	Hales	Derek	Nutrition	Other	view
	Smith	Falon	Center for Health Promotion and Disease Prevention	Other	view

External Institutions

Liaison	Last Name	First Name	Department Name	Role	
Drexel University					
★	Moore	Renee	Department of Epidemiology and Biostatistics	External Site PI	view

If your research includes personnel from a UNC Health Network Entity (NE), the UNC Health Office of Research Support and Compliance (ORSC) will review your IRB application and/or submitted UNC Health Collaboration Survey. You may be contacted by ORSC for additional information. IMPORTANT: In addition to obtaining IRB approval, you must also receive ORSC clearance for project personnel employed by the NE site(s). Project personnel MAY NOT proceed with research activities until you have obtained both approval from the IRB and clearance from the NE. Upon completed ORSC review, an ORSC NE Clearance Form will be provided and uploaded to the IRB application Study Documents section.

NOTE: The IRB database will link automatically to UNC Human Research Ethics Training database and the UNC Conflict of Interest (COI) database. Once the study is certified by the PI, all personnel listed (for whom we have email addresses) will receive separate instructions about COI disclosures. The IRB will communicate with the personnel listed above or the PI if further documentation is required.

3. If this research is based in a center, institute, or department (Administering Department) other than the one listed above for the PI, select here. Be aware that if you do not enter anything here, the PI's home department will be AUTOMATICALLY inserted when you save this page.

Department	Center for Health Promotion and Disease Prevention

3. Funding Sources

1. Is this project funded (or proposed to be funded) by a contract or grant from an organization EXTERNAL to UNC-Chapel Hill?

   Yes

Is UNC-CH the **direct** recipient of any Federal funding for this study? You should answer 'yes' *only* if you are the grantee. You should answer 'no' if you are the recipient of a sub-award or contractor under the grant.

Yes

Funding Source(s) and/or Sponsor(s): Please list all entities that are providing monetary support or supplies (e.g., study drug, gifts, devices at no cost, or others that provide in-kind services).

Sponsor Name	UNC Ramses Number	Sponsor Type	Prime Sponsor Name	Prime Sponsor Type	Sponsor/Grant Number	Detail
National Institutes of Health (NIH)	20-5519	Federal				view

2. Is this study funded by UNC-CH (e.g., department funds, internal pilot grants, trust accounts)?

   No

3. Is this research classified (e.g. requires governmental security clearance)?

   No

4. Is there a master protocol, grant application, or other proposal supporting this submission (check all that apply)?

- ✔ Grant Application
- ✘ Industry/Federal Sponsor Master Protocol
- ✘ Student Dissertation or Thesis Proposal
- ✘ Investigator Initiated Master Protocol
- ✘ Other Study Protocol

5. Is this a Clinical Study?
   Check YES if this study involves research using human volunteers that is intended to add to medical knowledge. There are two main types of clinical studies: clinical trials and observational studies. Do NOT check yes merely because you are conducting research in a clinical setting or using clinical data.
   Click here for additional definition of "Clinical Study"

   Yes

Does your study ONLY include observational activities?
Observational study: A type of clinical study in which participants are identified as belonging to study groups and are assessed for biomedical or health outcomes. Participants may receive diagnostic, therapeutic, or other types of interventions, but the investigator does not assign participants to a specific interventions/treatment. A patient registry is a type of observational study.

No

Will this clinical trial be listed in ClinicalTrials.gov, either by you or the sponsor?
Click here for ClinicalTrials.gov Guidance Information

Yes

Choose the appropriate Phase designation for this clinical trial.

- ✘ Pilot Study
- ✘ Phase 0
- ✘ Phase I
- ✘ Phase I/II
- ✘ Phase II
- ✘ Phase III
- ✘ Phase IV
- ✔ Other

If other, please explain
Behavioral intervention

4. Screening Questions

The following questions will help you determine if your project will require IRB review and approval.

The first question is whether this is RESEARCH (click for details)

1. Does your project involve a systematic investigation, including research development, testing and evaluation, which is designed to develop or contribute to generalizable knowledge? PLEASE NOTE: You should only answer yes if your activity meets all the above.

   Yes

The next questions will determine if there are HUMAN SUBJECTS (click for details)

2. Will you be obtaining information or biospecimens through intervention or interaction with the individual, and use, study, or analysis of the information or biospecimens? This would include any communication or interpersonal contact between investigator and subject such as using in-person or online questionnaires/surveys, interviews, focus groups, observations, treatment interventions, etc. PLEASE NOTE: Merely obtaining information FROM an individual does not mean you should answer 'Yes,' unless the information is also ABOUT them.

   Yes

3. Will you be obtaining, using, studying, analyzing, or generating identifiable private information or identifiable biospecimens collected through means other than direct interaction? This would include data, records or biological specimens that are currently existing or will be collected in the future for purposes other than this proposed research (e.g., medical records, ongoing collection of specimens for a tissue repository).
   OR
   Will you be using human specimens that are not individually identifiable for FDA-regulated in vitro diagnostic (IVD) device investigations?

   No

The following questions will help build the remainder of your application.

4. Will subjects be studied in the Clinical and Translational Research Center (CTRC, previously known as the GCRC) or is the CTRC involved in any other way with the study? (If yes, this application will be reviewed by the CTRC and additional data will be collected.)

   No

5. Does this study directly recruit participants through the UNC Health Care clinical settings for cancer patients **or** does this study have a focus on cancer or a focus on a risk factor for cancer (e.g. increased physical activity to reduce colon cancer incidence) **or** does this study receive funding from a cancer agency, foundation, or other cancer related group? (If yes, this application may require additional review by the Oncology Protocol Review Committee.)

   No

6. Is the UNC Chapel Hill IRB taking or being asked to take responsibility for the oversight of research by individuals, groups or organizations outside of UNC Chapel Hill? Or you are asking the UNC Chapel Hill IRB to cede review to an External IRB. If so, a reliance agreement will need to be executed prior to conducting any research activities.

   Yes

5. Multi-site Study Information

1. Is UNC-CH the Lead Site or Coordinating Center or Sponsor of a multicenter project?

   Yes

Lead Site/Coordinating Center addendum

The Lead Site/Coordinating Center addendum is not required if you are relying on an external IRB. In the attachments section, click Lead Site/Coordinating Center addendum and select the Not Yet Available / Not Applicable checkbox.

2. Is UNC-CH taking or being asked to take responsibility for the oversight of research by individuals, groups or organizations outside of UNC-CH?

   Yes

When a collaborator(s) outside of UNC-CH is (a) exercising authority or responsibility on behalf of a group or organization, (b) performing activities designated by a group or organization, or (c) using the collaboration for scholarly advancement (e.g., promotion, tenure) at a group or organization, complete the following information:

External Institution	Has or will the external institution agree to rely on the UNC-Chapel Hill IRB?	Local Consent Forms and Ads	Local Context Worksheet	Agreement
Drexel University	Yes	✗	✗	
**Personnel**	**Role**	**Ethics**	**CV**	**MD License**
Renee Moore	External Site PI	✗	✗	✗

View sIRB Attachments

Researchers are reminded that additional approvals may be needed from relevant "gatekeepers" to access subject.

Location

1. Are UNC-affiliated researchers involved in research conducted at any locations outside of the United States?

   No

Part A. Questions Common to All Studies

A.1. Background and Rationale

A.1.1. Provide a summary of the background and rationale for this study (i.e., why is the study needed?). If a complete background and literature review are in an accompanying grant application or other type of proposal, only provide a brief summary here. If there is no proposal, provide a more extensive background and literature review, including references.

Child care centers - and their providers - are critical partners in public health efforts to address today's obesity epidemic. Diet and physical activity (PA) habits are formed early, influencing immediate and long-term obesity risk, and both centers and providers play key roles in forming children's weight-related habits. Yet, the standard paradigm of child care-based obesity prevention initiatives primarily target only center directors to change organizational-level policies and practices; failing to recognize the needs of the child care providers. These providers suffer disproportionally high prevalence of obesity and research has shown that providers' own poor eating and PA behaviors reduce their confidence and ability to model and promote healthy lifestyle behaviors to the children in their care. However, information regarding the effectiveness of improving provider health behaviors or whether such improvements elicit meaningful change in the child care environment and the children in their care is limited. Leveraging our team's expertise with implementing evidence-based behavior change and weight loss interventions, the proposed study will integrate an evidence-based weight management intervention for child care providers into Go NAPSACC's existing childhood obesity prevention program (Go NAPSACC Enhanced). The Go NAPSACC Enhanced program will be assessed using a clustered randomized controlled trial to evaluate if improving child care providers' health behaviors elicit meaningful change in dietary and PA behaviors in 2-5-year-old children in their care and the child care environment. We will recruit 84 centers, including 168 providers, and 672 2–5-year-old children to participate in the evaluation. Centers will be randomly assigned to 1) standard "Go NAPSACC" or 2) "Go NAPSACC Enhanced" (with a provider weight management component). Outcome measures will

assess impact on dietary intake and PA behaviors of 2-5-year-old children at 6 months (primary aim) and 12 months. Secondarily we will compare the impact of the intervention on centers' implementation of healthy weight practices and the effect on provider-level weight, diet quality and/or PA at 6 and 12-months. Extensive process analysis, guided by the RE-AIM framework, will document the fidelity of the interventions, challenges and barriers to effective implementation, and use of program specified activities. If successful, findings will provide a highly implementable, scalable, and sustainable strategy that would enhance the standard paradigm of early childhood obesity prevention initiatives. See attached grant proposal for full background and rationale.

A.1.2. State the research question(s) (i.e., specific study aims and/or hypotheses).

Our **central hypothesis** is that meeting the interests and demands of providers, via a Go NAPSACC weight management program, will improve the health behaviors of the provider, subsequently boost instruction and modeling of these healthy lifestyle behaviors, and enhance Go NAPSACC's effect on the child care's use of healthy weight practices for the children in its care. Centers will be randomly assigned to 1) standard "Go NAPSACC" or 2) "Go NAPSACC Enhanced" (with a provider weight management component). Assessments will be completed at baseline, 6, and 12 months to address the following aims:

Aim 1. Improve dietary quality and PA behaviors of 2-5-year-old children.

H1a: Children enrolled at Go NAPSACC Enhanced centers will demonstrate greater improvements in diet quality and PA at 6 months relative to those in the standard Go NAPSACC arm (primary hypothesis).

H1b: Children enrolled at Go NAPSACC Enhanced centers will maintain greater improvements in diet quality and PA at 12 months relative to the standard Go NAPSACC arm.

Aim 2. Improve weight and weight related behaviors (dietary intake and PA) of child care providers.

H2: Providers enrolled at Go NAPSACC Enhanced centers will manifest greater improvements in weight, diet quality, and PA at 6 and 12 months relative to standard Go NAPSACC arm.

Aim 3. Improve the nutrition and PA environments at child care centers.

H3: Centers receiving Go NAPSACC Enhanced will make greater improvements in provisions, practices, and policies around children's healthy eating and PA at 6 and 12 months relative to standard Go NAPSACC.

A.2. Subjects

A.2.1. Total number of subjects proposed across all sites by all investigators (provide exact number; if unlimited, enter 9999):

924

A.2.2. Total number of subjects to be studied by investigators being provided oversight by the UNC IRB. (provide exact number; if unlimited, enter 9999):

924

A.2.3. If the above numbers include multiple groups, cohorts, or ranges or are dependent on unknown factors, or need any explanation, describe here:

Child care directors (n=84)

Child care teachers (n=168)

Children (n=672)

A.2.4. Do you plan to enroll subjects from these vulnerable or select populations:
If you will include children, prisoners or nonviable neonates or neonates of uncertain viability, please check the appropriate category below and complete the additional sections.

You should check "Pregnant women" if you specifically intend to recruit women who are pregnant or are not excluding pregnant women in biomedical research that is greater than minimal risk. Do not check if you are conducting a survey of the general public or conducting secondary data analysis or chart review not aimed at pregnant women.

Only check UNC-CH Student athletes, athletic teams, or coaches if you have specific plans to enroll these subjects. This is not applicable for intramural or club sports. For definitions and guidance see SOP 1201: Vulnerable subjects in research.

✔ Children (under the age of majority for their location)

Any minor subject who attains the age of majority during the course of the research study must provide consent as an adult, unless consent has been waived, which is requested in section D.3.1.

✘ Pregnant women

✘ Nonviable neonates or neonates of uncertain viability

✘ Prisoners, others involuntarily detained or incarcerated (this includes parolees held in treatment centers as a condition of their parole)

If an enrolled participant becomes incarcerated during the course of the research, they must be removed from the research project until such time as the IRB (and OHRP for NIH funded projects) approves the study to include prisoners, unless there is an immediate risk to the participant from ending treatments under the protocol.

✘ UNC-CH Student athletes, athletic teams, or coaches

A.2.5. Based on your recruitment plan and target sample population, are you likely to include any of the following as subjects? Select all that apply. This is not applicable to secondary data analysis or chart review.

Based on your responses, the consent form builder will insert the required text into your consent form template.

✘ Decisionally impaired individuals

(e.g., Mini mental state examination (MMSE), Montreal cognitive assessment (MOCA))

✘ Children who are wards of the State (Foster children)

✘ Non-English-speaking individuals

✘ UNC-CH Students

✘ UNC-CH Employees

✘ People, including children, who are likely to be involved in abusive relationships, either as perpetrator or victim.
This would include studies that might uncover or expose child, elder or domestic abuse/neglect. (See SOP Appendix A)

A.2.6. If any of the above populations are checked (excluding 'Decisionally impaired individuals' and 'Children who are wards of the State (Foster children)'), please describe your plans to provide additional protections for these subjects.

Given the young age of the child participants (2-5 years old), we will collect parental consent for child participation. Formal child assent is not appropriate given the young age; however, we will not force consented children to participate in any aspect of the study against their will.

A.2.7. Age range of subjects:

Minimum age of subject enrolled	2
	years
Maximum age of subject enrolled	99
» If no maximum age limit, indicate 99	
	years

A.2.A. Children

Research involving children (45 CFR 46 Subpart D or 21 CFR 50 Subpart D)

A.2.A.1. Why is it necessary to involve children as subjects for this research? If the study addresses a condition that particularly affects children, please explain.

The primary aim of this study is to improve the quality of dietary intake and physical activity behaviors of 2-5-year-old children. It is necessary to assess the effect of the intervention on the targeted audience.

A.2.A.2. Describe potential for direct benefit to children participating in this study OR if no prospect of direct benefit to children participating in this study, explain how research is likely to yield generalizable information about the condition. If applicable, please explain how benefit would differ for children randomized to active (i.e. treatment or intervention) versus placebo (i.e inactive or control) groups.

Child care center providers using Go NAPSACC may improve the diet quality and physical activity environment of their centers, which in turn may impact the diet quality and physical activity behaviors of the children at the center. As both intervention groups will have access to Go NAPSACC, both will have the opportunity to benefit children.

A.2.A.3. Describe the unique risks associated with children AND discuss your plans to minimize the risks and provide additional protections.

There are no additional unique risks for children.

A.3. Inclusion/exclusion criteria

A.3.1. List required characteristics of potential subjects (i.e., inclusion and exclusion criteria). If not covered, list also characteristics that would preclude their involvement.

Center eligibility criteria:

- Be open year-round
- Licensed with no plans to close in the next 2 years.
- Been in operation for at least one year.
- Have at least two classrooms with children 2-5 years old.
- Serve at least lunch to children ages 2-5 years old
- Have no history of Go NAPSACC participation in the past 6 mos.
- At least two 2-5-year-old classroom providers and 4 parents (within each classroom) must provide consent for the center to remain eligible for the study.

Providers eligibility criteria:

- Ability to provide informed consent
- Age = 18 yrs. old or older
- Child care teachers only: Be a teacher in a 2-5 year old classroom
- Child care teachers only: Not pregnant, not planning on becoming pregnant, and does not become pregnant during the study

Child Eligibility criteria

- In a classroom with a participating child care teacher

- 2-5 yrs. old
- Consenting primary caregiver must be able to read English

A.3.2. Justify any exclusion based on race, gender or ethnicity

N/A

A.3.3. Will pregnant women or women who become pregnant be excluded?

Yes

If yes, provide justification and describe the type and timing of pregnancy testing to be used:

It is not typically recommended that women lose weight during pregnancy. Instead, they encourage pregnant women to focus on getting enough nutrients and exercise to keep themselves healthy. Weight loss in those pregnant or wanting to become pregnant would require additional medical oversight beyond what is proposed in the present study.

A.4. Study design, methods and procedures

Your response to the next question will help determine what further questions you will be asked in the following sections.

A.4.1. Will you be using any **methods or procedures commonly used in biomedical or clinical research** (this would include but not be limited to drawing blood, performing lab tests or biological monitoring, conducting physical exams, administering drugs, or conducting a clinical trial)?

Yes

A.4.2. Describe the study design. List and describe study procedures, including a sequential description of what subjects will be asked to do, when relevant.

The proposed study will use a cluster randomized control trial to evaluate if improving child care providers' health behaviors elicit meaningful change in dietary and PA behaviors in 2-5-year-old preschool children in their care and the child care environment. To evaluate the impact, measurements will be collected at three-time points baseline, post-intervention (6 months), and maintenance (12 months post, no contact). Measures will be collected using a combination of self-report surveys, direct observation/measurement, and interviews by trained project staff. Additionally, The Go NAPSACC Enhanced arm will include a child care provider-level weight management intervention that encourages a reduced energy diet (stop light diet [SLD]), increased PA, and regular weight monitoring. We will use commercially available applications for self-monitoring PA (Fitbit Inspire 3) and weight (Aria wireless scales).

Randomization. Centers will serve as the unit of randomization. Following baseline testing of each center, the study statistician (Dr. Moore) will randomize centers to either standard Go NAPSACC or Go NAPSACC Enhanced. Randomization tables will be created by Dr. Moore using a permuted block approach, with block sizes of 2-4 to ensure equal distribution between arms throughout the randomization period. Dr. Moore will deliver intervention assignments directly to the study coordinator, who will inform participating centers and the Health Educator delivering the intervention. Investigators (except the study statistician) and data collectors will be kept blinded.

Intervention

Standard Go NAPSACC. Centers randomized to standard Go NAPSACC will receive the traditional Go NAPSACC program. Go NAPSACC is grounded in Social Cognitive Theory (SCT), targeting constructs (e.g., expectancies, observational learning, self-efficacy, behavioral capacity, environment, situation, reinforcement, reciprocal determinism) hypothesized to produce changes in the child care environment which foster healthier habits in enrolled children. The implementation model used to support center wide changes focuses on engaging center directors and orienting them to Go NAPSACC, giving access to interactive online tools that guide change, and providing ongoing support as they work to adopt organization-level evidence-based healthy weight practices. More specifically, the 6-mo. implementation model that will be employed in this trial will include:

- Director Engagement and Orientation. The director will sign on to lead the Go NAPSACC effort for their child care center, helping to ensure that changes are applied center wide and not just to specific classrooms. The directors will take part in a 1-hour, video conference orientation. The orientation will be provided by Go NAPSACC's Implementation Advisor using standardized materials, which include a slide presentation with talking points and a user guide. The orientation will instruct center directors on how to use the Go NAPSACC online tools and guide them through the creation of a personalized account. A 6-month timeline will also be provided that offers suggested milestones for completing each of the 5 steps.
- Access to Go NAPSACC Tools. Directors will use their personalized account to access Go NAPSACC's interactive, online tools that guide them through the 5-step improvement process: 1) assess, 2) plan, 3) take action, 4) learn more, and 5) keep it up. **Assess** tools provide self-assessments to help directors reflect on their current practices. **Plan** tools help directors use feedback from their self-assessment to select goals and create customized action plans to reach those goals. **Take action** tools offer a library of tips and materials that help directors as they work through their action plan steps. **Learn more** tools offer trainings to improve knowledge and skills for healthy weight practices. **Keep it up** tools encourage directors to retake the self-assessment to gauge progress and identify areas for future work.
- Ongoing Support. For 6 months, directors will receive monthly check-ins by telephone or email from the Implementation Advisor. Check-ins will be used to encourage directors to complete at least one cycle of Go NAPSACC's 5-step process (using milestones from the 6-mo timeline to guide pace), inquire about progress on goals, troubleshoot technical issues, and support navigation of the tips and materials library and trainings.

No contact follow-up (7-12 months). Centers will continue to have access to the Go NAPSACC online tools and will be encouraged to continue additional cycles of the improvement process; however, the active engagement of centers to provide ongoing support will cease. Because standard Go NAPSACC is delivered solely through interaction with center directors, there will be no direct contact between the research team and children or child care providers, except during data collection (0, 6, and 12 months).

Go NAPSACC Enhanced. Centers randomized to Go NAPSACC Enhanced will receive the traditional Go NAPSACC program (described above); in addition, child care providers will simultaneously receive an evidence-based weight management intervention. The Go NAPSACC Enhanced online tool created for providers will focus on personal weight management strategies. Each provider will have their own account (set up at orientation) through which they will take a self-assessment and choose a goal of weight maintenance or weight loss. Additionally, they will have access to materials that will support their adoption of evidence-based strategies for their weight management or loss goal. These strategies, also guided by SCT, pull from national nutrition and PA behavior recommendations and behavior change strategies that increase intervention adherence and improve weight loss. The intervention integrates techniques known to be most effective for changing diet and PA behaviors, including intention formation, goal setting (e.g., =5% weight loss at 6 months and no regain at 12 months), self-monitoring (diet, PA, weight), and feedback. These techniques will be enhanced by employing behavior shaping (gradually modifying diet and PA), stimulus control (strategies to decrease cues for less desirable and increase cues for more desirable diet and PA behaviors), including cognitive strategies to increase self-efficacy and to deal with social relationships affected by weight, such as identifying/confronting self-sabotage attempts, responding to stress with non-food techniques, and relapse prevention strategies to teach participants to recognize precursors and consequences of lapses, and developing plans for addressing high risk situations. The 6-months weight management intervention will include:

- Child Care Provider Orientation. Child care Providers will take part in a 1-hr., in-person orientation session, where a Health Educator will provide an introduction to Go NAPSACC Enhanced weight management intervention, including an overview of the Go NAPSACC Enhanced website and creation of a personalized account, a description of the self-assessment and choosing a weight management or loss goal, a detailed description of the diet and PA components, a 6-mo timeline with suggested milestones and instructions on self-monitoring devices. All participants will be provided with , a Fitbit Inspire 3TM activity tracker, and an Aria wireless scale (Fitbit, Inc., San Francisco, CA), and time will be allotted for demos, practice, questions, and troubleshooting (e.g., internet connectivity, app data entry). While providing these devices may somewhat diminish future scalability and sustainability, the

standardized devices are required for all participants to ensure connection (syncing Fitbit and wireless scales) and to allow investigators to push remote updates to devices. Participants will have full access to study related materials and commercial apps from their personal smartphones and computers. Any technical issues will be resolved by contacting project staff by phone or email.

- Dietary Modification. The reduced energy diet will follow the SLD approach (previously described) based on weight management goal (loss or maintenance). Starting weight will be used to suggest tailored red food limits: Weight Loss - <250lbs = 3; 250 - <300 = 4; >300 = 5. Weight Maintenance - <250 = 4; 250 - <300 = 5; >300 = 6. The SLD approach is suitable for all participants (including for whom weight loss may not be recommended [e.g., BMI <25 kg/m^2] as it does not specifically prescribe a calorie deficit but focuses on improvement of diet quality.
- *Physical Activity*. We will prescribe a progressive moderate intensity PA program (walking, jogging, biking, etc.) as recommended the "Physical Activity Guidelines for Americans" of 150 min/wk. Starting active minutes goal will be based on self-reported current activity levels: 0 min/wk = 10 min/day goal; 1-59 min/wk = 15/min/day goal, >60 min/wk = 20 min/day goal.
- *Self-monitoring* . Participants will be asked to self-monitor diet and PA daily, and body weight weekly. This data, which is automatically available to both participants and Health Educator, will be used for participant motivation and accountability, and to inform counseling by the Health Educator. *Diet.* Participants will log and view their dietary progress on the "Diet" page of the Go NAPSACC Enhanced website, which will include the current day's tally of caloric beverages and red foods, in addition to a graph of totals over time. *PA.* Participants will wear a Fitbit Inspire 3 wireless activity tracker on their non-dominant wrist. This device tracks PA with minimal participant burden and provides real-time data, via the Fitbit app. *Body weight*. Participants will be asked to weigh weekly using a Fitbit Aria wireless scale which interfaces with the Fitbit app to provide feedback to both participants and the Health Educator. These non-fasting weights will be used only for participant/Health Educator feedback, not as outcome weights.
- *Behavioral lessons*. The Go NAPSACC Enhanced website will unlock new modules that will convey the primary lesson objectives weekly during weight loss (0-6 months). Automated notifications of the addition of new modules will be sent to participants, via text message, including a link that opens directly to the Lessons webpage. Lessons will consist of written content focusing on reinforcing weight management strategies, including what strategies look like, why they are important, and practical tips for incorporating them into their everyday lives. Lessons will include topics such as portion sizes, reducing caloric beverages and red foods, emotional eating, behavioral strategies to increase PA, importance of sleep and stress management, stimulus control, sustaining changes, problem solving, and relapse prevention.
- Ongoing support. Participants will receive weekly reminders, via Twilio using encrypted text messaging, to complete self-monitoring. Participants self-monitoring data on diet, PA, and weight will be available immediately, but it will also be used to provide tailored weekly feedback. The weekly tailored feedback will be sent to participants via Twilio and offer congratulations for meeting goals, additional tips for those not meeting goals, or encouragement to monitor behavior for those who do not sync Fitbit and scale data. In addition, participants will also get weekly text messages through Twilio encouraging them to access the lessons (with quick access link). Participants will also receive motivational messages and tips for behavior change at random intervals each week, which has been shown to increase the effectiveness of SMS-based interventions. All messaging through Twilio is encrypted. Twilio encrypts data both in transit and at rest. They support TLS 1.2 to encrypt network traffic between participants and Twilio and Twilio data is encrypted at rest utilizing industry standard encryption algorithms.

Secondary users (any other center staff) from the Go NAPSACC Enhanced arm will have access to the website and its resources. No data will be collected on these users.

No contact follow-up (7-12 mos). Participants will continue to have access to Go NAPSACC Enhanced online tools, self-monitoring devices (i.e., tablet, Fitbit, wireless scale), and previously posted lessons and resources. They will be encouraged to continue self-monitoring diet, PA and weight. We will track the frequency of self-monitoring during the no-contact period; however, feedback from the Health Educator will not be provided and the active engagement of participants via self-monitoring reminders, tailored feedback, and motivational messages will cease.

Outcome Measures and Program Evaluation

Data Collection. Outcome measures will be collected on participating child care centers, providers, and children at three time points (0, 6, and 12 months). Although the Go NAPSACC program will be implemented center-wide (available to all children and child care providers in each center), measurements will only be collected on children and child care providers formally enrolled in the study. These measures will be collected in large part during a two-day on-site visit to the child care center and supplemented with self-administered surveys. This on-site visit will be conducted by data collectors who have undergone extensive training and certification on all measurement procedures and are blinded to study-arm assignment. Identical data collection protocols will be used at each timepoint.

In addition, interviews will be conducted by phone in a 20% random sample of child care directors and providers from the Go NAPSACC+ arm at 12 months to gather information that might be useful in improving the intervention, and/or implementing the intervention in the child care setting. Topics will include preference for intervention format, length, barriers to intervention components, suggestions to improve the intervention, overall satisfaction, including the diet and PA recommendations, online trainings, and Health Educator.

A.4.3. If subjects are assigned or randomized to study "arms" or groups, describe how they are assigned.

- *Describe the methods of computing the randomization schedule (if any) and maintaining blinding (if any).*
- *Who will perform these computations?*
- *How will you verify each subject's eligibility prior to randomization?*

Centers will serve as the unit of randomization. Following baseline testing of each cohort, the study statistician (Dr. Moore) will randomize centers to either standard Go NAPSACC or Go NAPSACC Enhanced. Randomization tables will be created by Dr. Moore using a permuted block approach, with block sizes of 2-4 to ensure equal distribution between arms throughout the randomization period. Dr. Moore will deliver intervention assignments directly to the study coordinator, who will inform participating centers and the Health Educator delivering the intervention. Investigators (except the study statistician) and data collectors will be kept blinded.

A.4.4. Describe any follow up procedures.

All measures will be collected at baseline, post-intervention (6 months), and maintenance (12 months post, no contact).

A.4.5. Once this study has been approved by the IRB, for how many months or years will this study be active (you are collecting data or have access to identifiers)?

Once approved, we anticipate the study being active from January 2022 through January 2027, or approximately 5 years. However, if additional data analyses are needed to fully interpret results, the length of the study could be extended. If we foresee such an extension needed, we will inform the IRB through a modification.

A.4.6. Will this study use any of the following methods?

- ✔ Audio Recording
- ✘ Video Recording
- ✔ Behavioral observation - (e.g., Participant, naturalistic, experimental, and other observational methods typically used in social science research)
- ✘ Pencil and paper questionnaires or surveys
- ✔ Electronic questionnaires or surveys
- ✘ Telephone questionnaires or surveys
- ✔ Interview questionnaires or surveys

- ✘ Other questionnaires or surveys
- ✘ Focus groups
- ✘ Diaries or journals
- ✘ Photovoice
- ✘ Still photography
- ✘ Unencrypted Messaging with Participants (e.g., text messages, unencrypted emails)

A.4.7. If there are procedures or methods that require specialized training, describe who (role/qualifications) will be involved and how they will be trained.

Training for the observation of center nutrition and physical activity policies and practices using the EPAO (environment and policy assessment and observation) will be provided by Regan Burney. Training will cover appropriate conduct and etiquette while at a child care center, review environmental components being assessed, and onsite practice and certification with one center.

Training on fitting accelerometers on children will be provided by Derek Hales. This training will also cover the proper use of the accelerometer.

Training on DOCC (diet observation in child care) protocol will be provided by Regan Burney. The training covers how to conduct observations of child dietary intake accurately and unobtrusively. Several hours of practice is incorporated to ensure that project staff can visually estimate amounts of foods and beverages served and consumed.

Training on height and weight will be provided by Derek Hales. This training will cover how to use standardized protocols to measure child height and weight, as well as tips for interacting with children during these measures to promote compliance.

Training for the process evaluation interviews will be provided by Regan Burney. This training will cover good interviewing techniques that will help the interviewer establish good rapport with the subject and consistently use open-ended questions and probes appropriately.

A.4.8. Are there cultural issues, concerns or implications for the methods to be used with this study population?

No

A.4.A. Biomedical methods and procedures

A.4.A.1. Is this an interventional study?

No

Distinguish what is being done specifically for this research from procedures that would be done anyway for clinical care:

No Answer Provided

A.4.A.2. If the study involves the use of placebo control, provide justification

N/A

A.4.A.3. Will this study involve drugs, biologics or other substances (such as a botanical or dietary supplement)?
For guidance on dietary supplements, see Section VI, C FDA guidance document UCM229175.pdf

No

A.4.A.4. Is there an Investigational New Drug application (IND) for this study?

No

Please check below:

✔ This study does not involve drugs, biologics or other substances.

✘ I am using a U.S. commercially available agent, consistent with labeling.

✘ I am studying a botanical substance or dietary supplement intended to affect the structure and/or function of the body; it is **not** intended to cure, treat, mitigate, prevent or diagnose disease, including its associated symptoms.

A.4.A.5. When the intent of a clinical investigation is to collect information about the safety or effectiveness of a device, the need for an Investigational Device Exemption (IDE) must be evaluated. Please review the Investigational Device Guidance document prior to completing this section. Your response to the following questions will determine if an IDE is needed.

A. Select the response that best describes your investigation:

✘ 1. This research is **investigator-initiated** and is designed to study one of the following:

- An unapproved device (includes assays [e.g. in vitro diagnostics], software, algorithms and some mobile applications.)
- An approved device with unapproved components
- A new indication for an approved device **even if no marketing application is planned**

✘ 2. This research is designed to support an IDE (device marketing application).

✘ 3. This research is designed to collect safety and/or effectiveness information about a device.

✔ 4. The device(s) in this research is being **used as "tool"** to address a research question, collect information or test a physiologic principle. No data is collected about the device itself.

✘ 5. None of the above.

Upload information about the device in the Attachments section. Include device description, brochures, illustrations, operating or procedural manuals, instructions/directions and anything else that may be helpful to the IRB in conducting a risk/benefit analysis.

A.4.A.6. Does your study involve any of the following? (check all that apply)

✘ Embryonic stem cells

✘ Fetal tissue

✘ Genetic testing (see GINA and GWAS)

✘ Clinical laboratory tests

If McLendon Labs will do the testing, you must complete the appropriate form found at UNC Health Care and submit to them for review.

✘ Testing for communicable diseases that have mandated reporting requirements (link to state guidance)

✘ Point of Care Testing (POCT), which is CLIA-approved testing done at the "bedside" or site of care by hospital or clinic personnel (not by subject). Examples include urine pregnancy testing, glucose monitoring, etc.

If McLendon Labs will do the testing, you must complete the POCT form found at UNC Health Care and submit to them for review.

✘ If your study utilizes **radiopharmaceuticals** to address basic science questions, an IND is not necessary.
Instead, your study will be reviewed/approved by the Radioactive Drug Research Committee (RDRC); approval by the Radiation Safety Subcommittee (RSS) is not required.
If you have questions about the RDRC approval process, please contact Dede Corvinus.

✖ Diagnostic or therapeutic ionizing radiation, or radioactive isotopes (not covered under 21 CFR 361.1), which subjects would not receive otherwise if not participating in this research study. Do not check if all radiation is administered as standard of care. Do check if your study includes views/scans that represent no greater than minimal risk as determined by the Radiation Safety Sub-committee (Guidance).
Application for Human Use of Radiation in Research.

Select which option applies to your study:	--

✖ Gadolinium administered as a contrast agent

✖ IBC (Institutional Biosafety Committee) - Recombinant DNA or gene transfer to human subjects

✖ Any research activities conducted in the UNCHC Perioperative areas. This includes Pre-care, Pre-op, Operating room and PACU.
You must complete the Checklist for Perioperative Services and return it to moe_lim@med.unc.edu

✖ Any form of medical imaging (ultrasound, MRI, CT, X-ray, PET-CT, PET-MRI)

A.4.A.7. Will your study involve storage of specimens for future unspecified research?

No

A.5. Benefits to subjects and/or society

A.5.1. Describe how this study will contribute to generalizable knowledge that will benefit society.

This trial is the first to test if a comprehensive early childhood obesity prevention initiative that also addresses health of providers elicits meaningful change on the health behaviors of the children in their care and the child care environment. If successful, findings will provide a highly implementable, scalable, and sustainable strategy that would enhance the standard paradigm of early childhood obesity prevention initiatives.

A.5.2. Does this study have the potential for direct benefit to individual subjects in this study?

Yes

Consider the nature, magnitude, and likelihood of any direct benefit to subjects. If there is no direct benefit to the individual subject, say so here and in the consent form, if there is a consent form. Do not cite monetary payment or other compensation as a benefit.

Explain

Go NAPSACC and Go NAPSACC Enhanced should lead to improvements in centers' adoption of best practices around children’s healthy eating and physical activity. Additionally, center staff in the Go NAPSACC Enhanced arm will learn about the benefits of physical activity and healthy eating and ways to improve physical activity and diet quality. We expect that this information will help workers become more informed decision-makers about their overall health. Additionally, by offering health promotion activities in their centers, worker satisfaction may increase, which may also increase employee retention. For the children enrolled at these child care centers, these efforts will likely lead to healthier environments that foster and support healthy eating and physical activity behaviors. Participation in this study could potentially lead to improved diet and physical activity behaviors, and consequently, improved health outcomes for children.

A.5.3. Are there plans to communicate the results of the research OR results of any clinical tests administered for the research back to the subjects?

Yes

If yes, describe

It is important to share study results with center staff to demonstrate the value of participating in research studies. It is our intention, therefore, to share a summary of the study's results with participating center staff. We would develop a brief summary (~2 pages) of study results reporting on the impact of both programs on physical activity and health behaviors. We would limit data used to only the first

two time points: baseline and post-intervention. Timing for distribution will require that necessary data are available from all cohorts, hence the earliest these would be available would be fall 2025. Additionally, a center would not receive their copy of this report until maintenance measures for that center are complete, hence distribution for centers starting in later waves would be delayed beyond winter 2026.

A.6. Risks and measures to minimize risks

For each of the following categories of risk you will be asked to describe any items checked and what will be done to minimize the risks.

A.6.1. Psychological

- ✔ Emotional distress
- ✔ Embarrassment
- ✔ Consequences of breach of confidentiality (Check and describe only once on this page)
- ✘ Other

A.6.2. Describe any potential psychological risks checked above and what will be done to minimize these risks

Risks of participation in the study are minimal. To minimize risks of emotional distress and embarrassment, project staff will emphasize to participants that there are no "right or wrong" answers to any questions. Additionally, participants will be assured that they are free to refrain from answering any question(s) that they find objectionable and that they may conclude their participation at any time. Furthermore, no information about specific health conditions will be asked. Participants will be assigned an ID to help protect their confidentiality and only this ID will appear on data collection instruments.

A.6.3. Social

- ✘ Loss of reputation or standing within the community
- ✘ Harms to a larger group or community beyond the subjects of the study (e.g., stigmatization)
- ✘ Consequences of breach of confidentiality (Check and describe only once on this page)
- ✘ Other

A.6.4. Describe any potential social risks checked above and what will be done to minimize these risks

Not applicable.

A.6.5. Economic

- ✘ Loss of income
- ✘ Loss of employment or insurability
- ✘ Loss of professional standing or reputation
- ✘ Loss of standing within the community
- ✘ Consequences of breach of confidentiality (Check and describe only once on this page)
- ✘ Other

A.6.6. Describe any potential economic risks checked above and what will be done to minimize these risks.

Not applicable.

A.6.7. Legal

- ✗ Disclosure of illegal activity
- ✗ Disclosure of negligence
- ✗ Consequences of breach of confidentiality (Check and describe only once on this page)
- ✗ Other

A.6.8. Describe any potential legal risks checked above and what will be done to minimize these risks

Not applicable.

A.6.9. Physical

- ✗ Medication side effects
- ✗ Pain
- ✓ Discomfort
- ✓ Injury
- ✗ To a nursing child or a fetus (either through mother or father)

A.6.10. Describe any potential physical risks checked above, including the category of likelihood and severity, and what will be done to minimize these risks. Where possible, describe the likelihood of the risks occurring, using the following terms:

- Very Common (approximate incidence > 50%)
- Common (approximate incidence > 25 - 50%)
- Likely (approximate incidence of > 10 - 25%)
- Infrequent (approximate incidence of > 1 - 10%)
- Rare (approximate incidence < 1%)

Describe severity of risks using the following grading scale:

- Mild- No disruption to the subject’s ability to perform daily activities; may include non-prescription intervention only
- Moderate- Temporary interference with daily activities; may include prescription intervention
- Severe- Interference with daily activities; medically significant but not life threatening
- Life threatening

Examples:

Rare (< 1%) and Severe: blindness
Rare (< 1%) and Mild: dry skin, dry mouth, transient headache

If you are using these terms differently than described above, please provide your study-specific definitions.

Phase 1 trials: Due to limited experience, incidence may be better described as the number of events that have occurred in the total number of animals/humans studied.

Increasing one's physical activity, particularly for individuals who are inactive, can be accompanied by some initial discomfort and soreness of muscles. Additionally, injury is possible as the result of participation in physical activity. For adult participants it will be recommended that they consult their primary care physician before making major changes to their physical activity habits.

A.6.11. Unless already addressed above, describe procedures for referring subjects who are found, during the course of this study, to be in need of medical follow-up or psychological counseling

Not applicable.

A.6.12. Are there plans to withdraw or follow subjects (or partners of subjects) who become pregnant while enrolled in this study?

Yes

If yes, explain

It is not typically recommended that women lose weight during pregnancy. Instead, they encourage pregnant women to focus on getting enough nutrients and exercise to keep themselves healthy. Weight loss in those pregnant or wanting to become pregnant would require additional medical oversight beyond what is proposed in the present study.

A.7. Data and safety monitoring

A.7.1. When appropriate, describe the plan for monitoring the data to ensure the safety of participants. These plans could range from the investigator monitoring subject data for any safety concerns to a sponsor-based data and safety monitoring board or committee (DSMB, DSMC, DMC), depending on the study. For studies that do not raise obvious safety concerns, you may still describe your plans for monitoring the study as it progresses.

Qualifications and responsibilities of the Safety Officer

The safety officers for this trial will be Truls Østbye, MD, MPH (primary) and Katelyn Holliday, PhD (associate). Dr. Østbye has an MD degree, experience running intervention studies, and has previously worked with our research team as a DSO on several of our completed childcare-based health promotion trials. Dr. Holliday has experience with health behavior interventions, particularly studying physical activity and cardiovascular disease relationships. As Safety Officers, Drs. Østbye and Holliday will review the reports sent by the study coordinator (at the frequency outlined below) and will use the checklist attached to this document to determine whether there is any corrective action, trigger of an ad hoc review, or stopping rule violation that should be communicated to the study investigator, the University of North Carolina-Chapel Hill IRB, and the NIDDK.

Measurement and reporting of subject accrual, adherence to inclusion/exclusion criteria

The Project Managers will track subject accrual throughout each cohort of recruitment. A recruitment tracking database will be created in order to monitor what happens with each center identified for the initial recruitment pool – completion of screening, eligibility or reasons for ineligibility, completion of consent, parent-child dyads and providers enrolled for each, and completion of measures. These data will be compiled bi-weekly and reviewed with the PI to ensure that the project is on target for enrolling sufficient numbers and that enrolled centers, providers, and children meet eligibility criteria. To help keep the DSO apprised of recruitment, updates (informal) will be shared with the DSO monthly.

Formal subject accrual reports will be generated at the end of each cohort of recruitment and shared with the PI and DSO. If concerns arise about failure to recruit sufficient numbers of centers or children, the PI, Co-Investigators, DSO, Project Managers will convene to discuss options for enhancing recruitment strategies.

Measurement and reporting of participant compliance to treatment protocol

The Project Manager and Interventionist will track all participant withdrawals, including the number, type, and reason for withdrawals. During follow-up, retention data will be reviewed at least biweekly with the PI.

Retention reports will be created at the conclusion of each cohort's post-intervention data collection and shared with the PI and DSO. If concerns arise about whether withdrawals and loss to follow-up has reached a level that might inhibit the ability of the study to test its primary hypotheses, the PI, Co-Investigators, DSO, Project Managers, and Interventionist will convene to discuss methods for preserving study power.

Below is an outline for a safety report for this trial.

I. Table of Contents

II. Narrative/ Trial Summary
- A. Summary of Main Findings
- B. Discussion of Issues or Problems
- C. Report Preparation Procedures

III. Study Description
- A. Project Organizational Chart, Personnel
- B. Brief Statement of Purpose of Trial
- C. Projected Timetable and Schedule

IV. Study Administration
- A. Recruitment Status
  1. Enrollment by Month
  2. Comparison of Targeted to Actual Enrollment

B. Retention Status
1. Overall Subject Status
2. Individual Subject Status

V. Study Data Reports/Tables or Figures
- A. Generic Information

1. Recruitment, Screening and Enrollment (Table 1)

2. Participant Characteristics (Table 2, 3, and 4)

3. Retention (Table 5)

4. Participation (Table 6 and 7)

5. Withdrawals (Table 8)

B. Safety Assessment

1. Adverse Events (Table 9)

2. Analysis (Table 10 and 11)

A.7.2. If not already addressed above, describe the plans for aggregate review of unanticipated problems (including but not limited to adverse events) across all sites, in order to monitor subject safety.

At enrollment, we will inform child care centers and their participating providers to contact the project manager if they feel an injury or illness may have occurred to them or a child in their care as a result of their participation in this study. If such an event occurs, the project manager will collect a detailed description of the event, the adverse outcome, severity of the adverse event, and whether or not participants viewed it as related to the study. This report will then be reviewed by the DSM officer, who will categorize it as: definitely unrelated, or unlikely, possibly, probably, or definitely related to study participation. Although injuries could occur as a result of an intervention that promotes regular physical activity, in our previous center-based work, no injuries related to participation were reported. We therefore anticipate that injury risk is present, but

minimal.

Adverse event reports will be submitted to the IRB after completion by the DSM officer. The PI will submit a summary of these reports to the NIDDK project officer on an annual basis. As a minimal risk study that complies with all guidelines for data and participant safety, study suspension is not anticipated. However, if such an event were to occur, the Principal Investigator would contact NIDDK immediately to report the suspension, give reasons for the suspension, and describe the plan to meet the requirements for lifting the suspension

A.7.3. What are the criteria that will be used to withdraw an INDIVIDUAL SUBJECT from this study or halt the research intervention (e.g., abnormal lab tests, allergic reactions, failure or inability to comply with study procedures, etc.)?

There are no instances where we would withdraw an individual subject from the study or halt the research intervention.

A.7.4. Are there criteria that will be used to stop the ENTIRE STUDY prematurely (e.g., safety, efficacy, unexpected adverse events, inability to recruit sufficient number of subjects, etc.)?

Yes

Please explain

Given the minimal risk associated with this intervention study, it is highly unlikely that an accumulation of excess adverse events would prompt stopping of the trial. However, as outlined elsewhere, we will monitor adverse event rates in all participants, and the DSO, together with the study investigators, will alert the IRB and the sponsor if a larger than reasonably expected injury rate should occur in the treatment group. Other issues relating to stopping rules for this trial include:

New Information

It is exceedingly unlikely that any new information comes available during this trial that would necessitate stopping the trial.

Limits of Assumptions

Difficulty in recruiting or retaining adequate numbers of participants could potentially require stopping the study. While protocols make every effort to avoid such issues, stopping rules have been created in case such an issue arises. Those rules are as follows:

failure to recruit adequate numbers of participants (red zone classification), or
failure to retain adequate numbers of participants (red zone classification)

Suboptimal recruitment is classified as falling into one of three zones – yellow, amber, and red. Red zone classification is the most severe of these categories and is defined as less than 25% of the benchmark at the 25% time point or the 50% time point, or less than 50% of the benchmark at the 75% time point.

It is possible that excessive study dropouts and/or missing data will limit the value of data analysis of measurements. In the grant proposal, we allowed for a 20% attrition in the recruited child care centers and children. Pre-post-test differences will be evaluated in order to examine the effects on the study power and to detect differences in the primary outcome. Results will be summarized and communicated to the PI, Co-Investigators, DSO, and NIDDK.

A.7.5. Will this study involve a data and safety monitoring board or committee?

No

A.8. Data analysis

A.8.1. Summarize the statistical analysis strategy for each specific aim.

Analytical Plan.

Statistical analysis. Data analyses will be performed using SAS statistical software (Version 9.4 or higher, Cary, NC). Baseline descriptive characteristics will be summarized globally, and by intervention group, using frequencies and percentages for categorical variables, and means and standard deviations (median [Q1, Q3] for non-symmetric data) for inherently quantitative variables. Prior to conducting data analyses, we will audit the data for completeness and quality, including missing data. We will evaluate distributions to ensure that they meet assumptions of planned analyses (see below), including the detection of outliers.

Baseline equivalence. As outlined in the consort statement extension for cluster RCTs, random assignment by individual ensures that any differences in group characteristics at baseline are the result of chance rather than some systematic bias; however, in cluster RCT the risk of chance imbalance is greater as clusters rather than individuals are randomized. Therefore, to assess the degree to which randomization resulted in equivalent groups, we will assess if baseline variables are distributed differently between intervention groups Baseline equivalence of student characteristics will be examined in two- and three-level hierarchical linear models, as appropriate, with an indicator for treatment arm. Baseline characteristics that demonstrate group nonequivalence will be controlled for in the analytic models.

Analysis of primary and secondary outcomes. Our primary analyses for Aim 1 will involve testing change in minutes of non-sedentary activity and HEI scores between standard Go NAPSACC and Go NAPSACC Enhanced groups at 6 (primary outcome) and 12 months (secondary outcome). Using maximum likelihood methods, we will use multi-level linear mixed models (PROC MIXED) with repeated measures to estimate change at 6 and 12 months and to test for statistical differences across groups in changes over time. Models will include random effect for cluster to account for covariance between participants within the same center as well as fixed effects for time, trial arm, time*arm interaction. A proper error covariance structure will be chosen based on model fit indicated by model likelihood, Akaike Information Criterion and Bayesian Information Criterion. Residuals will be examined to check the assumptions of models. The results from the mixed models will be summarized with adjusted means and 95% confidence intervals (CI) by trial arm at each time point. To further explore the effect of the intervention, these analyses will be followed by sensitivity analyses that adjust for baseline variables distributed differently between intervention groups and examine completers only. Similar analyses will be completed for each secondary outcome: 1) provider weight, 2) provider device measured MVPA (Actigraph), 3) provider HEI score (ASA24), and 4) overall EPAO nutrition and PA environment scores. We will also use multi-level linear mixed models to examine secondary outcomes as longitudinal covariates and asses their association with longitudinal measures of primary outcomes, controlling for treatment, at 6 and 12 months.

Missing data and statistical models. All analyses will be conducted using the intention-to-treat principle (ITT), in which all available data on all randomized participants are included. This approach minimizes bias if individuals drop out of the trial for different reasons. Every effort will be made to obtain follow-up data on all patients randomized, whether or not they follow their assigned treatment. Our maximum likelihood approach assumes that any missing outcome data is missing at random (i.e., missing data including that due to drop-out can be dependent on any previously observed outcomes or treatment assignment). With this approach, we use all data that have been collected without regard to whether data are missing for a patient at another visit, including drop-out, and without explicit imputation of missing data. If the combined rate of missingness on variables is above 5%, we will consider imputing missing endpoint data using multiple imputation techniques and will assess the sensitivity of our results to various assumptions of missing data patterns.

A.8.2. If this is a pilot study, please describe the future study and say how its study design, aims, sample size, and methods differ from the pilot study you are proposing.

This is not a pilot study.

A.8.3. Provide a compelling justification for the proposed sample size in terms of the likelihood of achieving each aim.

Power analysis. This trial is powered to detect between group difference (Standard Go NAPSACC vs. Go NAPSACC Enhanced) of 5 units in HEI score and 1 minute per hour in non-sedentary PA. The sample size and power calculations for the proposed cluster RCT design depends on the following parameters: the number of child care centers, the number of students per school (~8; ~4 children x 2 classrooms), effect size (d), provider-level and center-level intra-class correlations (ICCs; ?), and the proportion of explained variance in school means by center-level covariates (R2).204 We anticipate that on average about 8 children and 2 providers will participate at each child care center, and conservatively calculated sample size estimating 0% of variance can be accounted for by covariates. The ICCs observed in our previous work assessing preschool children's dietary intake and PA (R01 HL120969) were 0.04 and 0.07 for HEI score and 0.07 and 0.10 for non-sedentary PA at the provider- and center-levels, respectively. Based on previous studies at child care, the standard deviation of change in HEI score is estimated at 11 units166,198 and non-sedentary PA is estimated at 3 minutes per hour.155,199 Furthermore, we calculated power for each of the two primary comparisons using a type I error rate of 2.5%, thus keeping overall Type I error rate at 0.05. Sample size was initially calculated using assumptions associated with non-sedentary PA knowing that the higher ICC would require a larger sample size. This sample size was then used to conduct a power analysis for HEI score.

Using this information and Optimal Design 3.01,205 a sample size of 84 centers, with two 2-5-year-old classroom per center, and an average of 4 children per classroom, for a total of 672 children would allow us to detect a clinically meaningful difference in non-sedentary PA with 88% power at a=0.025 level of significance. This sample size also provides more than 99% power to detect a difference of 5 units in HEI score between the two study arms. Even with up to 20% attrition for centers and children, a completer's only analysis will still provide 81% and 98% power for change in non-sedentary PA and HEI score, respectively, which is higher attrition than we expect based on our previous trials.130,155,202 Based upon our sample size for our primary aims, we have included effect sizes that we will be powered to detect for our secondary outcomes with 80% power.

A.8.4. Summarize the plans for data management.

Participants will be assigned an ID number as part of the screening process. All data collection forms and tables thereafter will use only this participant ID. Participant information (ID, eligibility/ineligibility, enrollment) will be stored in a database on UNC Center for Health Promotion and Disease Prevention's secure SQL. This server is only accessible to approved project staff. Within this master database, fields with individually identifying information are encrypted and decrypted only for display on-screen.

Trained and certified project staff will be responsible for collecting study outcomes data. Data will be collected using confidential online Qualtrics self-report surveys and paper forms. All data collection instruments will use only the participant's assigned ID number and only necessary project personnel will have access to Qualtrics project account. Any paper forms will be brought to UNC offices immediately and the UNC Project Manager will check completed surveys for completeness and accuracy. All paper forms will be scanned for accuracy and completeness by the Project Manager and be entered and saved to shared UNC data server to create electronic files. All data will be cleaned and processed by the data manager (Hales) using recommended procedures. The UNC Project Manager (Burney) will oversee quality checking of all data, creation of derived variables, development of a data dictionary and data user manual, and the creation of the final master database.

All paper documentation will be stored in a locked file cabinet in the offices at UNC. The project managers at UNC will be responsible for keeping cabinet keys stored securely and limiting access to only appropriate study staff. Offices are also locked automatically after normal business hours.

All aspects of recruitment screening, intervention delivery, and data collection will be the responsibility of the investigators and project staff and UNC, while data analysis will be led by the study statistician at Drexel. The master database and any electronic records of recruitment or outcomes data will be kept on a secure server at UNC, with password-restricted access. The master database will be managed by the UNC Project Manager

(Burney), and access will be limited to key study personnel. The UNC Project Manager will manage the project folder (and all electronic files within) on the secure shared drive (OneDrive), which will be accessible only to registered key personnel (UNC and Drexel study statistician), in accordance with an agreed upon Data Use Agreement. UNC will be responsible for summarizing recruitment and enrollment information for the data safety monitoring report using criteria specified in the data safety monitoring plan.

A.9. Identifiers

A.9.1. Check which of the following identifiers you already have or will be receiving, or select "None of the above."

- ✔ Names (this would include names/signatures on consent forms)
- ✔ Telephone numbers
- ✔ Any elements of dates (other than year) for dates directly related to an individual, including birth date, admission date, discharge date, date of death. For ages over 89: all elements of dates (including year) indicative of such age, except that such ages and elements may be aggregated into a single category of age 90 and older
- ✔ Any geographic subdivisions smaller than a State, including street address, city, county, precinct, zip code and their equivalent geocodes (e.g. GPS coordinates), except for the initial three digits of a zip code
- ✘ Fax numbers
- ✔ Electronic mail addresses
- ✘ Social Security numbers
- ✘ Medical record numbers
- ✘ Health plan beneficiary numbers
- ✘ Account numbers
- ✘ Certificate/license numbers
- ✘ Vehicle identifiers and serial numbers (VIN), including license plate numbers
- ✘ Device identifiers and serial numbers (e.g., implanted medical device)
- ✘ Web universal resource locators (URLs)
- ✘ Internet protocol (IP) address numbers
- ✘ Biometric identifiers, including finger and voice prints
- ✘ Full face photographic images and any comparable images
- ✘ Any other unique identifying number, code, or characteristic, other than dummy identifiers that are not derived from actual identifiers and for which the re-identification key is maintained by the health care provider and not disclosed to the researcher
- ✘ None of the above

A.9.2. For any identifiers checked, how will these identifiers be stored in relationship to the research data?

- ✔ with the research data (i.e., in the same data set and/or physical location)
- ✔ separate from the research data (i.e., coded with a linkage file stored in a different physical location)

Provide details about the option you selected above:
Center director, providers, and parent emails, telephone numbers, and addresses will be collected in order to communicate with them about the relevant project components (e.g., data collection, site visits, implementation, interviews). All electronic data will be stored on a secure server, and all paper forms/data will be stored in locked cabinets in locked office suites. Electronic files and paper forms with identifying data (e.g., emails and telephone numbers) and consent forms will be stored separately from the rest of study data and forms. A master identification key will be created but stored separately from study data. Electronic files and paper forms with identifying data will be destroyed one year following completion of the main study.

A.9.3. Are you collecting Social Security Numbers to be used as a unique identifier for study tracking purposes for national registry or database? (Do not check yes if collecting SSN *only* for payment purposes; this will be addressed later.)

No

A.10. Confidentiality of the data

A.10.1. Describe procedures for maintaining confidentiality of the data you will collect or will receive (e.g., coding, anonymous responses, use of pseudonyms, etc.).

A number of steps will be taken to protect center staff against any possible risks, including breaches of confidentiality. First, outcomes data will be collected by trained and experienced personnel with clearly established data collection procedures. All study personnel will also be required to complete the human subjects' protection certification, as well as the good clinical practice for social/behavioral research certification available at the University of North Carolina-Chapel Hill (CITI Training). Data collection trainings will reiterate the importance of protecting subjects against potential risks and maintaining confidentiality of all data.

Additionally, collection of data is for research purposes only, and is kept in strict confidence by study personnel. To help protect confidentiality, all participants will be assigned a numeric ID. All data collection tools will use that ID in order to minimize the appearance of identifiers (e.g., subject name) on paper forms and in electronic data tables. Hard copies of forms will be stored in a locked file cabinet at the UNC Center for Health Promotion and Disease Prevention.

Electronic data files will be stored primarily on HPDP's secure password-protected server. Since CHAI is assisting with several components of the study, including web-based intervention components (e.g., goal selection, weekly behavior tracking, and weekly tailored feedback), some data will also be located on CHAI's secure server. Both HPDP and CHAI administration policies and programming coding standards are in compliance with current UNC security regulations. To ensure data security of web-based data, the UNC network utilizes a secure authentication mechanism, the Transport Layer Security (TLS) system, which is the successor to the Secure Sockets Layer (SSL) protocol. This security mechanism supports encryption in both directions (to and from the website) and protects your username and password and also protects your data with network encryption. Since TLS is web-based, it provides encryption of data across all devices including mobile devices such as smartphones. The Go NAPSACC website is password protected allowing users to recover and update their password in circumstances where their smartphones have been lost or stolen.

The ASA24 is an automated self-administered 24-hour dietary assessment tool through the National Cancer Institute (NCI). Participants are assigned a username and password by the study team (this username is not related to the person at all and will be a variation of the project name, e.g., gnscares1) and no identifying information will be asked by the ASA24. Per their Researcher Site Agreement, the researcher is responsible for directing human subjects under its supervision to submit data to the NCI. NCI will store the data in NCI's ASA24 database. NCI will maintain the data in the absence of identifying information; however, NCI will have the right to assign a unique alphanumeric username tag to records describing individual human subjects. NCI shall ensure that the nature of the such ta will prevent NCI from determining human subject identities. The researcher, but not NCI will retain the crosswalk for mapping the tag to identify individual human subjects under the researcher's supervision. NCI will not have access to the crosswalk. NCI will not communicate with any human subjects providing data.

Data submitted to the NCI is property of the NCI for distribution purposes and shall be made available as a

service to research, academic, and clinical communities. The data will only be used for research, clinical and academic purposes.

Only the PIs, project managers and appropriate study staff will have access to identifying information. Once consents are obtained and participant ID assigned, only the PIs and project manager will have access to the consent forms.

Participants in the Go NAPSACC Enhanced arm will receive messaging through the Go NAPSACC Cares website, using Twilio. All messaging through Twilio is encrypted. Twilio encrypts data both in transit and at rest. They support TLS 1.2 to encrypt network traffic between participants and Twilio and Twilio data is encrypted at rest utilizing industry standard encryption algorithms.

A.10.2. Describe how data will be transmitted among research team (i.e., personnel listed on this application).

As noted above, the raw data are stored on the UNC Center for Health Promotion and Disease Prevention's secure password-protected SQL server, with pieces, also being stored on CHAI's secure server. Access to these data will be limited to approved project personnel. Approval for access is controlled by the Principal Investigator (Willis) and Project Manager, with the assistance of our IT contact, Scott Wilber. The data manager (Hales) will have access to these data and be responsible for the data management. In preparation for analysis, the data manager will generate a specific time-point de-identified dataset. This de-identified dataset will be saved on shared drive on UNC's secure server. Those wanting access to the analysis dataset will request permission from the PI and project manager, who in turn will work with the data manager to extract the specific data requested (de-identified). Any transfer or copying of data files to another computer or server would comply with standards set forth by UNC Information Security Policy.

A.10.3. Are you collecting sensitive information such as sexual behavior, HIV status, recreational drug use, illegal behaviors, child/physical abuse, immigration status, etc?

No

A.10.4. Do you plan to obtain a federal Certificate of Confidentiality for this study? Please note that all ongoing or new research funded by NIH as of December 13, 2016 that is collecting or using identifiable information is automatically issued a Certificate of Confidentiality (CoC). You should also select "Yes" if your study is NIH funded and has been issued a CoC under this updated NIH policy.

NOTE: Investigators utilizing ANY federal funding to conduct this research should review the COC website to determine if their funding agency issues COCs automatically (as the NIH does) or if they might need to apply for the COC via the online COC system. Unfunded and non-federally funded investigators may also apply for a COC via the online COC system.

Yes

A.10.5. If this study is limited to data collection by survey or interview, discuss the potential for deductive disclosure (i.e., directly identifying subjects from a combination of indirect IDs).

Not applicable

A.10.6. Will any of the groupings or subgroupings used in analysis be small enough to allow individuals to be identified?

No

A.11. Data sharing and transmission

A.11.1. Check all of the following who will receive **identifiable data** (contains any of the 18 identifiers listed above) outside the immediate research team (i.e., not listed as personnel on this application)? *

- ✔ No one
- ✘ Coordinating Center
- ✘ Statisticians

- ✗ Consultants
- ✗ Other researchers
- ✗ Registries
- ✗ Sponsor and/or its designee(s)
- ✗ External labs for additional testing
- ✗ Journals
- ✗ Publicly available dataset
- ✗ Other

A.11.2. For any recipients checked above, explain the confidentiality measures to be taken

No Answer Provided

A.12. Post-study disposition of identifiable data or human biological materials

A.12.1. Describe your plans for disposition of data or human biological specimens that are identifiable in any way (directly or via indirect codes) once the study has ended. If you plan to destroy linkage codes or identifiers, describe how and when this will be done.

Study data will be maintained by ID throughout the study period in a locked file cabinet and locked office and on a secure password-protected server. These data will be maintained in this manner throughout the study period and for 2 years after the study has ended. An extension of the IRB protocol may be sought for additional data analysis following the active study period. Once the active protocol has ended or at the end of 2 years (whichever comes later), all identifiable data will be destroyed using appropriate confidential records management procedures.

Part B. Direct Interaction

B.1. Methods of recruiting

B.1.1. Check all the following means/methods of subject recruitment to be used:*

- ✓ In person
- ✗ MyChart

To utilize MyChart for research recruitment purposes, please complete the form (click here), and upload a PDF copy of the completed MyChart request to your application.

- ✗ Participant pools
- ✗ Presentation to classes or other groups
- ✗ Letters
- ✓ Flyers
- ✗ Radio, TV recruitment ads
- ✗ Newspaper recruitment ads
- ✗ Website recruitment ads
- ✓ Telephone script
- ✓ Email or listserv announcements
- ✗ Follow up to initial contact (e.g., email, script, letter)
- ✗ N/A
- ✗ Other

✘ Social Media

B.1.2. **Research for Me @UNC**
A comprehensive study listing and engagement site intended to fulfill the mission of improving transparency and awareness of research at UNC.
All study involving direct interaction with participants must be listed on this site; you may choose whether to further utilize your listing for participant recruitment.
Instructions:

- Choose Basic or Recruitment
- Click on link to open listing submission form in a new tab
- Submit online form

✔ **Basic Listing** **(Click here to open basic submission form)**

For studies that do not want to be contacted by potential participants. Submit very basic information in lay language, but no details or team contact information will be displayed.
Exception from this kind of listing is rare, but may be requested for consideration via this form.

✘ **Recruitment Listing** **(Click here to open recruitment submission form)**

For studies that want to utilize the free recruitment features of the website. Participants can view more details about your study and contact the team to express interest.
You control the timeframe for display. Get a unique URL and QR code for use on other materials. Site is promoted to patients and the public by NC TraCS.

View examples, manage submitted listings, find FAQ, and download PDFs at researcherdashboard.unc.edu
Please direct all questions and feedback to Research for Me

B.1.3. Describe how subjects will be identified

Because this is a child care-based intervention, we will recruit child care centers, then staff from those centers, and then children within enrolled classrooms. We will be identifying child care centers within a 100 mile radius of the UNC-Chapel Hill. Each cohort will target a specific 1-4 county area. Potential child care centers will be identified from the publicly available online database of licensed child care programs provided by the NC Division of Child Development. The database includes child care center's name, business address, phone number, and email address (if available). This database allows for identification of licensed programs by type of facility (e.g., center), and by location (e.g., county), functions which will be used to identify a pool of centers specific to our counties of interest. Directors of these centers will be invited to take part in the study and we will work with those interested and eligible center directors to distribute information about the project to their center staff and parents.

B.1.4. Select any of the following procedures solely conducted for screening, recruiting, or determining the eligibility of prospective human subjects. (Note: you should only collect the minimal information needed for these purposes.)

✔ Obtain information through oral or written communication with the prospective subject or legally authorized representative
This includes online, telephone, or in-person screening questionnaires or interviews.

✘ Obtain already collected identifiable private information or records
Examples include review of medical charts, data repositories, and administrative records.

✘ Reviewing/testing identifiable biospecimens by accessing stored biospecimens and related information

✘ None of the above

B.1.5. For any selections made, please describe the procedures. (Respond “N/A” if “None of the above” is selected.)

A trained research staff member will call center directors and use a screening script to screen for interest and eligibility. For teachers and parents of children a research staff member will visit the center and use a screening script to screen participants for interest and eligibility.

B.1.6. For any information collected for these purposes, please describe when and how you will destroy the data if the participant declines to participate or is not eligible. (Respond “N/A” if “None of the above” is selected.)

If a participant is screened as ineligible or uninterested, their data will be stored separately from enrolled participants. These data will be kept until the end of the study to fully track and document reasons for ineligibility, and frequency of disinterest resulting in screen failures. These data are necessary to accurately describe the recruitment process in the consort diagram. These data will be destroyed with the rest of the study data one year after the study is completed.

B.1.7. Describe how and where subjects will be recruited and address the likelihood that you will have access to the projected number of subjects identified in A.2.

We will use multiple strategies to recruit eligible child care centers identified from the online database. First, potentially eligible child care center directors for whom we have an email address will receive an e-invitation (i.e., Email Invite) with a brief introduction to the study. The email will include contact information for the project office, a link to the study recruitment website, and a recruitment flyer (i.e., Director Informational Flyer) as an attachment. We will follow-up with a mailed invitation (i.e., Mail Invite). Similar to the email invitation, the mailed invitation will provide contact information for the project office, the web address of the recruitment website, and a recruitment flyer.

We will follow-up these invitations with recruitment phone calls, conducted by trained study staff using the Recruitment and Screening Script. If the director is unable to talk when initially called or they request time to think about whether or not they want to participate, the study staff member will schedule a time to call back. If the center director expresses interest, the study staff member will screen the director to confirm center eligibility. If eligible, a study staff member will set up a time for the study staff member to visit and answer any questions the director has then and to visit with potential 2-5 year old teacher participants. During this initial recruitment call, potential eligible 2-5 year old teachers will be identified and directors will be asked to talk to the teachers before the planned visit. Following the call a welcome email will be sent to the director. Strategies used have been developed in collaboration with our Community Advisory Committee and used successfully in previous studies recruiting child care centers in North Carolina.

The welcome email will include a link to an online consent form and demographics (a copy of the consent form will be attached). Additionally, a flyer they can show the teachers will be attached and guidance will be provided on how to share the information with teachers. A recap of the director being eligible and interested will be in the email as well as a reminder that the research staff member will visit during the agreed upon day and time at the center where they are happy to answer any questions. Directors will be encouraged to call or email the project office if they have any questions about the study.

Information about the study will also be distributed via local professional meetings, community events, and regular newsletters/mailings/communications from community partners (e.g., local Smart Start partnerships). For these communications, we will use a combination of the Flyers and Email Announcement.

Once a center director agrees to participate we will set up a time to visit the center to talk with potentially eligible 2-5 year old classroom teachers during nap/break time. During this time a research staff member will use the Teacher Recruitment Script to assess interest and eligibility. We will also provide the same flyer previously emailed to the center director. A copy of the consent form will be brought for teachers to look through and keep as a copy. Finally, if a teacher is eligible and interested the research staff member will provide a Flyer for parents to put on the door, welcome packets to put in each child's cubby/folder, guidance on how to talk to parents about the project, and set up a time to return to the classroom to recruit children. After the visit is complete, a recap email will be sent to each teacher as well as a link to a consent form if needed and a reminder of the day/time a research staff member will return for the visit.

For recruitment of children, a research staff member will visit the participating 2-5 year old teacher's classroom during general pick up time. At this time the staff member will pick up and note any completed consent and demographic forms parents filled out prior to the visit. Otherwise, the research staff member will talk with parents of eligible children to gain interest using the Parent Recruitment Script. In classrooms where more than 4 children volunteer to take part in measures, we will use random selection to choose study participants.

Secondary users, staff from participating child care centers not participating in measurement, will be given access to the Go NAPSACC Enhanced website and its resources after the participating teachers have completed orientation to Go NAPSACC Enhanced. Directors will be emailed a link for secondary users to sign up and then they will be given access to the website. No outcome assessments will be collected on secondary users.

Please note that all photographs, interviews, footage, etc. included in project/recruitment materials and video are photographs and/or footage taken for the project for which we have obtained signed photo/video releases or purchased stock photos authorized for public dissemination.

B.1.8. Describe how you will protect the privacy of potential subjects during recruitment

All recruitment materials will provide an email address and telephone number that will allow them to contact the project office directly. Project staff who field these incoming contacts work in an office space dedicated to this project to ensure participant privacy. The email address supplied is directed to a password protected email account and the telephone number is
directed to a dedicated project line with a password protected voicemail account. Only designated project staff will have access to the email account or voicemail account.

Recruitment efforts may also include a site visit to the child care center, during which project staff will make themselves available in the participating teacher's classroom only to answer questions about the project from potential participants. Potential participants will be encouraged to contact the project office if they have any questions or would prefer the opportunity to speak with someone about the project with greater privacy.

Results of recruitment and screening will be tracked electronically. This screening log will be stored on the secure server, which only appropriate project team members will have access to.

B.1.9. Describe how subjects will be contacted, if not addressed above

How subjects will be contacted is addressed above.

B.1.10. Describe who (by role) will do the recruiting

Recruitment for the main study will be led by the Project Manager (Regan Burney) with support from research study staff. Updates regarding project personnel who will assist with the main study will be submitted to IRB as part of a subsequent modification.

B.1.11. Describe efforts to ensure equal access to participation among women and minorities

It is our intention to include a good representation of women and minorities in our sample. We anticipate that child care providers will be predominately women given that the child care workforce in North Carolina is 99% female. We anticipate that approximately 50% of the children will be girls, as there is roughly equal participation in child care across the sexes.

B.2. Protected Health Information (PHI)

Protected Health Information (PHI) is any identifiable information about the subject's health that relates to their participation in this research and is obtained from sources other than the subject, such as medical records, health care providers, insurance plans, etc. more

B.2.1. Are you requesting a limited waiver of HIPAA authorization?
If you need to access Protected Health Information (PHI) to identify potential subjects who will then be contacted, you will need a limited waiver of HIPAA authorization (see SOP 1801, 2.3). This does not apply to situations where you will never contact subjects directly (e.g., retrospective chart review), in which case you should request a full waiver under section D.

No

B.2.2. Will you need ongoing access to PHI (e.g., medical records) to conduct the study beyond identification of potential subjects as addressed above OR will you need to retain PHI obtained for screening? In this case, you will need to obtain a signed HIPAA Authorization from each subject.

No

B.3. Subject Contact, Duration and Privacy

B.3.1. Number of contacts per subject (contacts includes in-person, telephone, email, mailings, etc.)

~81

B.3.2. Duration of each contact. If multiple contacts, provide the range or average time for each contact.

The number and duration of contacts will vary by the type of participant (child care center director vs. Worker vs Parent/child dyads). Most contacts (72) will be for delivering of campaign messages, which include prompts for weekly monitoring of behaviors and receipt of tailored feedback. Active intervention lasts 24 weeks, with 3 contacts per week, each contact lasting about 5-10 minutes. There will also be 6 contacts with center directors for technical assistance webinars, each lasting about 30-60 minutes. There will also be up to 3 contacts during each data collection period. Additional contacts may be needed as full details regarding intervention and control programs are decided during the project planning phase. Final protocols will be submitted to IRB for approval in a subsequent modification.

B.3.3. Total duration of individual subject's participation, including follow up evaluation, if applicable

12 months

B.3.4. Where are you studying subjects or obtaining their data?

✔ Non-healthcare setting
✘ Healthcare setting

B.3.5. Describe procedures that will ensure privacy of the subjects in this study. Examples include the setting for interviews, phone conversations, or physical examinations; communication methods or mailed materials (e.g., mailings should not indicate disease status or focus of study on the envelope)

Recruitment phone calls will be conducted in private offices with the door closed. Surveys will be distributed online as often as possible, giving participants the option to complete them in private. Additionally, if paper copies are required or asked for, participants will be given ample time away from research staff members to complete in private. Follow-up interviews with participants will be conducted in a private office with the door closed and if Zoom is used cameras will be turned off.

B.3.6. Provide more information about the location(s) where research will be conducted (e.g., if UNC Medical Center is checked in #4 above and study visits will be conducted in the CTRC, enter "CTRC" here.)

Project activities will take place at multiple locations. Recruitment calls will take place at the Center for Health Promotion and Disease Prevention. Recruitment visits and data collection will take place in each child care center. Children will be measured primarily at the child child care center. Individuals filling out online surveys and tracking of behaviors will be online and the participants will be able to select a location (e.g., home, work) where they feel comfortable submitting and receiving this information.

B.4. Incentives for participation

B.4.1. Are there incentives (monetary or non-monetary) for subjects to participate or are you reimbursing subjects for study-related costs (e.g., travel, parking, hotel accommodations or childcare)?

Yes

A. Please describe any incentives and/or reimbursements for study-related costs separately below.

Centers will receive $100 for participating in the 2-day onsite data collection visit at baseline, $100 at post-intervention, and $100 at follow-up. Total $300 per center.

Child care providers will receive $75 for data collection at baseline, 6, and 12 months to compensate for time. Total of $225 per teacher over 3 time points.

Participants in Go NAPSACC Enhanced will be given a Fitbit Inspire 3, an Aria Air wireless scale, and other small items complimentary to the lessons of the program (e.g., water bottle, exercise bands, cooling towel) at the go NAPSACC Enhanced orientation soon after baseline measurement is completed.

Participants in the control arm (standard Go NAPSACC) will receive financial incentives equal approximately to the cost of intervention materials ($225) as compensation for not receiving the intervention which will be distributed equally at each time point (baseline, 6 months, 12 months).

A random sample of 20 Go NAPSACC Enhanced participants will be chosen to participate in structured interviews. Each participant will receive $25 for their time to complete the structured interviews at post-intervention

Children will receive small gift items valued up to $3 per child (e.g., chalk, bubbles, books) for data collection at baseline, 6, and 12 months to compensate for time. Additionally, when minimal number of children are consented in each classroom (at least 4 children) classrooms will receive items to enhance their classroom environment (e.g., poly spots, play kitchen food) to thank parents and teachers for their time.

B. Specify the schedule for incentives and if/how this will be prorated if the subject withdraws (or is withdrawn) from the study prior to completing it.

Incentives described will be for the completion of key measures at each of the three measurement time points described- baseline, post-intervention, and maintenance. Those who wear and return their accelerometer, allow for study staff to complete anthropometric measurements, and complete and return surveys will receive the full incentive. Those who do not participate in measurements or provide only partial data will not be incentivized for that measurement time point. Participants in the control arm (standard Go NAPSACC) will receive financial incentive soon after the completion of baseline measures, equal approximately to the cost of intervention materials ($225) as compensation for not receiving the intervention and devices. This will be equally distributed at each time point.

C. For compensation in foreign currency, provide a US dollar equivalent.

N/A

D. Discuss the potential for coercion, given factors like the amount of the incentive, the age of the subjects, the purchasing power in foreign countries, the time involved and complexity of procedures, etc.

We do not anticipate any potential for coercion given that the level of incentives as planned are not excessive. The maximum individual incentive at any point in time is expected to be $150. As small to medium size businesses, the maximum center incentive of $100 is not viewed as a large or coercive amount of money.

E. If the subjects are children who will receive the compensation, i.e., the child, the parents or both?

Children

B.4.2. Are you collecting Social Security numbers or ITIN for payment and/or tax-related purposes?

No

B.5. Costs to be borne by subjects

B.5.1. Will there be any costs that subjects will incur related to participation in the study? Do not include costs for standard care for which patients would be billed if they were not in this study. Also do not include the time spent participating in the study.

No

Part C. Existing Data, Records, Specimens

C.1. Data Sources

C.1.1. What existing records, data or human biological specimens will you be using? (Indicate all that apply or select 'None of the above'):

- ✗ Medical records in any format.

  ALERT: You must check both boxes: 1) Medical records in any format and 2) Electronic medical record using Epic, or you/your study team will not be granted access to Epic for research purposes.

- ✗ Electronic medical records using Epic, WebCIS or other electronic system
- ✗ Carolina Data Warehouse for Health (CDW-H) (for UNC and its affiliates only)
- ✗ Carolinas Collaborative Data Request and Review Committee (DRRC)
- ✗ Paper medical records

If you access the medical records of fewer than 50 patients under a full or limited waiver of HIPAA, submit a copy of your IRB approval letter and a completed Research Disclosure Form to Health Information Management (HIM). Do not submit this information to the IRB. For additional information about this process, you should contact HIM directly at : 919-595-5591 or 919-966-1225 or 919-595-5580.

- ✗ Data already collected from another research study

Were the investigators for the current application involved in the original collection?	--

- ✗ Patient specimens (tissues, blood, serum, surgical discards, etc.)

Has the clinical purpose for which they were collected been met before removal of any excess?	--

- ✗ Data already collected for administrative purposes
- ✗ Student records (You will need to satisfy FERPA requirements: see SOP 3101, section 3.1 for guidance)
- ✗ UNC Dental Records
- ✗ Data coming directly from a health plan, health care clearinghouse, or health care provider?
- ✓ Publicly available data
- ✗ Other
- ✗ None of the above

For EACH data source checked above, provide a description of the data, proposed use, how data were collected (including consent procedures), and where data currently reside.

Data from an online database of licensed child care facilities maintained by the NC Division of Child Development and Early Education will be used to identify child care centers to be contacted during recruitment. https://ncchildcare.ncdhhs.gov/childcaresearch

C.1.2. Describe your plans for obtaining permission from the custodians of the data, records or specimens (e.g., pathology dept, tissue bank, original researcher):

N/A - this is a publically available database.

C.1.3. Do the custodians of the data, records or specimens require a data use agreement?

No

C.2. Coding and Data Use Agreements

C.2.1. When you receive these data, records or human biological specimens will they be coded? Coded means identifying information that would enable the research team to readily ascertain the individual's identity has been replaced with a number, letter, symbol, or combination thereof (i.e., a code). If you will not be using existing materials, check "No."

No

Part D. The Consent Process

D.1. Obtaining informed consent from subjects

The standard consent process is for all subjects to sign a document containing all the elements of informed consent, as specified in the federal regulations. Some or all of the elements of consent, including signatures, may be altered or waived under certain circumstances. If you will be requesting a waiver answer "not applicable" for any of the following questions that will not pertain to this study. You will be asked to provide relevant information in the section below on waivers.

D.1.1. Will children under the age of majority in their locale (18 years in NC) be enrolled? (Note: Any minor subject who attains the age of majority during the course of the research study must provide consent as an adult, unless consent has been waived, which is requested in section D.3.1.)

Yes

Explain the process for obtaining consent from the subject, parental permission and/or minor assent as applicable (unless a waiver of permission will be requested later) in the sections below. The informed consent process should include the following:

- Provide the participant/parent/LAR with:
  - Information about the study in a language they understand
  - An opportunity to ask questions and have their questions answered
  - Adequate time to consider study participation
  - A signed copy of the consent form (a copy is acceptable)
- Avoid exculpatory language and undue influence.
- Document the consent process in the research record; if consent takes place on the same day as study procedures, document that informed consent was obtained prior to initiating any research-related procedures.

When explaining the process for obtaining consent/assent below, please incorporate the above information. (e.g., do not simply state that the participant will sign the form). The assent process should be developmentally appropriate and provide opportunities for children to discuss their willingness or unwillingness to participate. If assent is required, a child's dissent (unwillingness to participate) MUST be honored.

Parents

Once a teacher agrees to be a part of the project, consent forms will be a part of the parent packet, which will be left in the child's cubby or folder. Parents will have ample time to look over in the privacy of their home. In order for their child to participate, a parent must give permission. We will include an attestation to that fact in the consent. Research study staff will come back to visit the center approximately 2-3 days later to explain the project further to parents and answer any questions. If still interested parents will be able to sign a consent form and the study staff will provide them with a copy for them to keep for their records.

Check the characteristics of children to be enrolled: *

✔	0 - 6 years
✘	7 - 14 years
✘	15 - 17 years

Explain the process for obtaining the assent of the child (unless waiver of assent will be requested, in which case you should provide justification here).

Given the young age of the child participants (2-5 years old), we will collect parental permission for child participation. Formal child assent is not appropriate given the young age; however, we will not force participating children to participate in any aspect of the study against their will.

D.1.2. Will adult subjects be enrolled in your study?

Yes

Explain the process for obtaining consent from the subject (unless a waiver of permission will be requested later) in the sections below. The informed consent process should include the following:

- Provide the participant/LAR with:
  - Information about the study in a language they understand
  - An opportunity to ask questions and have their questions answered
  - Adequate time to consider study participation
  - A signed copy of the consent form (a copy is acceptable)
- Avoid exculpatory language and undue influence.
- Document the consent process in the research record; if consent takes place on the same day as study procedures, document that informed consent was obtained prior to initiating any research-related procedures.

When explaining the process for obtaining consent/assent below, please incorporate the above information. (e.g., do not simply state that the participant will sign the form).

Center directors and staff

We will have two different consent forms based on whether the participant is a center director or teacher. Once an individual has been deemed eligible to participate in the study, study staff will email individuals and direct them to a unique Qualtrics link (sent via email) to an online informed consent. After clicking on the link, they will be directed through a series of screens that represent the informed consent document. There is evidence that informed consent provided online does not differ from that obtained in person and, with certain techniques built in to the system such as the need to scroll down to get to the next page and timers to ensure adequate time on each screen, may actually result in better comprehension of the study over using a single paper consent document.

In order to participate, the study participant (not a legally authorized representative) must give consent. We will include an attestation to that fact in the consent. Once participants have read the consent form, they will be asked to click either a button to signify their consent ("I have read the information and voluntarily agree to participate in this research study" or not (“I do not wish to participate in this research study.”) Individuals that consent to participate will be provided with a link of the consent form they can download or print for their records. Individuals that do not consent will be directed to a page that thanks them for their time and provides contact information for study staff in the event that they are interested in participating but have questions about the consent form or study.

D.1.3. Will decisionally-impaired subjects be enrolled in your study? (includes unconscious patients, some psychiatric disorders, others who lack the capacity to give consent)

No

D.1.4. Are you planning to obtain consent from any Non-English speaking subjects?

No

D.1.5. Describe who (by role) will be obtaining consent or parental permission.

Consent will be obtained by trained study staff members supervised by the Project Manager.

D.1.6. Discuss the potential for influencing the subject's decision to participate. Describe steps that will be taken to minimize undue influence during the consent process. These might include a waiting period between the initial consent discussion and obtaining consent, or obtaining consent by someone other than a person with perceived authority (e.g., professor, employer, treating physician).

Child care center staff and parents will have multiple days from the time they hear about the study until a consent form needs to be signed. This will give them ample opportunity to look over materials and ask questions to study staff before signing. Study protocols and training will ensure that study staff will not coerce any potential center staff into the study.

D.1.7. Has the sponsor of this study provided a model consent form?

No

D.2. Waiver of written documentation of informed consent

The default is for subjects to sign a written document that contains all the elements of informed consent. Under limited circumstances, the requirement for a signed consent form may be waived by the IRB. For example, this might occur for phone or internet surveys, when a signed consent form is either impractical or unnecessary, or in circumstances where a signed consent form creates a risk for the subject.

D.2.1. Are you requesting a waiver of any aspect of written (signed) documentation?

No

D.3. Full or partial waiver of consent

The default is for subjects to give informed consent. A waiver might be requested for research involving only existing data or human biological specimens. More rarely, it might be requested when the research design requires withholding some study details at the outset (e.g., behavioral research involving deception). In limited circumstances, parental permission may be waived. This section should also be completed for a waiver of HIPAA authorization if research involves Protected Health Information (PHI) subject to HIPAA regulation, such as patient records.

D.3.1. Are you requesting any of the following:

✗ a waiver of informed consent in its entirety

✗ a waiver or alteration of some of the elements of informed consent

✗ a waiver of HIPAA authorization (If you are accessing patient records for this research, you must also request a waiver of HIPAA authorization)

D.3.2. If your request for a waiver applies to some but not all of your subject groups and/or consent forms, please describe and justify

No Answer Provided

D.3.3. Does this request for waiver support a study design that involves deception or withholding of information?

No

Consent Forms

This submission requires the following consent forms

Template Type
Adult Consent Form
Parental Permission Form

This submission includes the following consent forms

File Name	Document Type
GNSCares_Director_Consent_Formv1.docx	Adult Consent Form
GNSCares_Teacher_Consent_Formv1.docx	Adult Consent Form
GNSCares_Parent_Permission_Formv1.docx	Parental Permission Form

view consent forms

Attachments

This submission requires the following attachments

Document Type
Grant Application
Lead Site/Coordinating Center addendum<br>**This attachment not provided because:** Not Yet Available / Not Applicable
Observation Guide
Electronic Questionnaire Survey<br>**This attachment not provided because:** Not Yet Available / Not Applicable
Interview Questionnaire Survey<br>**This attachment not provided because:** Not Yet Available / Not Applicable
Device Description
Flyer for Recruitment
Telephone Script for Recruitment<br>**This attachment not provided because:** Not Yet Available / Not Applicable
Email or Listserv Recruitment<br>**This attachment not provided because:** Not Yet Available / Not Applicable

This submission includes the following attachments

File Name	Document Type
GNS_WM_Full_Proposal_2021.02.26.docx	Grant Application
manual_inspire_3_en_US.pdf	Device Description
Emails_Content _GNS Cares.docx	Email or Listserv Recruitment
GO NAPSACC CARES Parent Flyer (Print).pdf	Flyer for Recruitment
GO NAPSACC CARES Teacher Flyer (Print).pdf	Flyer for Recruitment
GO NAPSACC Cares Center Flyers.pdf	Flyer for Recruitment
GNC invitation 4.75 x 6.5 (print).pdf	Other Materials for Recruitment
Recruitment and Screening Scripts_GNS Cares_11.10.22.docx	Telephone Script for Recruitment
GNScares_Survey_CHD_Demographics_DRAFT+RB.docx	Electronic Questionnaire Survey
GNScares_Survey_CNT_Demographics_DRAFT.docx	Electronic Questionnaire Survey
Teacher surveys combined.pdf	Electronic Questionnaire Survey
Teacher_diet_asa24-overview-2020-02232021.pdf	Electronic Questionnaire Survey
GNS+_Structured Interview Guide_IRB.docx	Interview Questionnaire Survey
EPAO_DOCC_protocolandforms_combined.pdf	Observation Guide
Anthropometrics_Provider_v1.pdf	Other Questionnaire Survey
Protocol-Addendum-Coordinating-Center-Lead-Investigator_Drexel.docx	Lead Site/Coordinating Center addendum
Go NAPSACC Enhanced Intervention Sample Content - Combined.pdf	Other
Go NAPSACC Intervention Sample Content - Combined.pdf	Other

view attachments

Addenda

Data Security Requirements

view addenda
