# Supplementary material for: “My wellbeing-their wellbeing “– An eHealth intervention for managing obesity in early care and education: Protocol for the Go NAPSACC Cares cluster randomized control trial
Source: PLoS One. 2023 Jul 7;18(7):e0286912. doi: 10.1371/journal.pone.0286912 (PMC10328321; doi:10.1371/journal.pone.0286912)
Supplement: Peer review history

# Editor report: Decision Letter 0

**DOI:** 10.1371/journal.pone.0286912.r001

**Authors:** Lucinda Shen

26 May 2023

“My wellbeing-their wellbeing “– an eHealth intervention for managing obesity in early care and education: Protocol for the Go NAPSACC Cares cluster randomized control trial.

PONE-D-23-12551

Dear Dr. Willis,

We’re pleased to inform you that your manuscript has been judged scientifically suitable for publication and will be formally accepted for publication once it meets all outstanding technical requirements.

Within one week, you’ll receive an e-mail detailing the required amendments. When these have been addressed, you’ll receive a formal acceptance letter and your manuscript will be scheduled for publication.

An invoice for payment will follow shortly after the formal acceptance. To ensure an efficient process, please log into Editorial Manager at http://www.editorialmanager.com/pone/, click the 'Update My Information' link at the top of the page, and double check that your user information is up-to-date. If you have any billing related questions, please contact our Author Billing department directly at authorbilling@plos.org.

If your institution or institutions have a press office, please notify them about your upcoming paper to help maximize its impact. If they’ll be preparing press materials, please inform our press team as soon as possible -- no later than 48 hours after receiving the formal acceptance. Your manuscript will remain under strict press embargo until 2 pm Eastern Time on the date of publication. For more information, please contact onepress@plos.org.

Kind regards,

Lucinda Shen, MSc

Staff Editor

PLOS ONE

Journal requirements:

When submitting your revision, we need you to address these additional requirements.

1. Please ensure that your manuscript meets PLOS ONE's style requirements, including those for file naming. The PLOS ONE style templates can be found at 

https://journals.plos.org/plosone/s/file?id=wjVg/PLOSOne_formatting_sample_main_body.pdf and 

https://journals.plos.org/plosone/s/file?id=ba62/PLOSOne_formatting_sample_title_authors_affiliations.pdf

Additional Editor Comments (optional):

Reviewers' comments:

# Editor report: Acceptance letter

**DOI:** 10.1371/journal.pone.0286912.r002

**Authors:** Lucinda Shen

31 May 2023

PONE-D-23-12551 

“My wellbeing-their wellbeing “– An eHealth intervention for managing obesity in early care and education: Protocol for the Go NAPSACC Cares cluster randomized control trial. 

Dear Dr. Willis:

I'm pleased to inform you that your manuscript has been deemed suitable for publication in PLOS ONE. Congratulations! Your manuscript is now with our production department. 

If your institution or institutions have a press office, please let them know about your upcoming paper now to help maximize its impact. If they'll be preparing press materials, please inform our press team within the next 48 hours. Your manuscript will remain under strict press embargo until 2 pm Eastern Time on the date of publication. For more information please contact onepress@plos.org.

If we can help with anything else, please email us at plosone@plos.org. 

Thank you for submitting your work to PLOS ONE and supporting open access. 

Kind regards, 

PLOS ONE Editorial Office Staff

on behalf of

Miss Lucinda Shen 

Staff Editor

PLOS ONE
